# Silicon Carbide Nanowires Impair Mucociliary Clearance-Mediated Innate Immunity in Primary Human Bronchial Epithelial Cells

**DOI:** 10.1021/acsnano.5c01981

**Published:** 2025-06-06

**Authors:** Ziting Wang, Jimmy Vernaz, Nikolaos Tagaras, Bernadett Boda, Tina Buerki-Thurnherr, Giacomo Reina, Vera M. Kissling, Samuel Constant, Govind Gupta, Peter Wick

**Affiliations:** † Nanomaterials in Health Laboratory, Department of Materials Meet Life, 111825 Swiss Federal Laboratories for Materials Science and Technology (Empa), St. Gallen 9014, Switzerland; ‡ Epithelix Sàrl, 18 chemin des Aulx, 1228 Plan-les-Ouates, Switzerland

**Keywords:** advanced materials, graphene, inhalation exposure, respiratory mucus, primary human bronchial epithelial cell culture, ciliopathy, fibrosis

## Abstract

The respiratory tract possesses mucociliary-driven innate immune defense mechanisms that protect the lungs from harmful environmental exposures, but when damaged, increase susceptibility to respiratory infections and diseases. Inhalation exposure to certain nanomaterials has been shown to trigger fibrosis and other respiratory conditions. However, there is a limited understanding of whether nanomaterials can impair mucociliary defense in lungs and its underlying mechanism. Here, we first investigated the fate of zero-dimensional, one-dimensional, and two-dimensional silicon- and carbon-based nanomaterials (silicon carbide nanowires (SiC NWs), silicon dioxide (SiO_2_), multiwalled carbon nanotubes (MWCNTs), and graphene nanosheets) in airway mucus. The results demonstrated that only SiC NWs escaped through the mucus gel without interactions, suggesting their potential to diffuse across the protective mucus layer. The hydrophobicity of the SiC NWs, associated with the low abundance of polar surface groups, such as silanols, was mainly responsible for the observed shielding of particle interactions with mucus components. Furthermore, repeated exposure to SiC NWs in primary bronchial epithelial cell cultures revealed abnormal ciliary structure and significantly (*p* < 0.05) compromised mucociliary clearance functions, however, no such effects were evident for other particles. mRNA expression analysis showed a significant (*p* < 0.05) increase in *FOX-J1* transcripts, suggesting transcriptional dysregulation of ciliogenesis after exposure to SiC NWs. Finally, SiC NWs reduced epithelial barrier integrity and promoted pro-inflammatory and pro-fibrotic responses. These findings unravel the hazardous potential of SiC NWs upon inhalation exposure and identify the breaching and impairment of the mucociliary innate defense as a key event in their respiratory toxicity.

## Graphical abstract

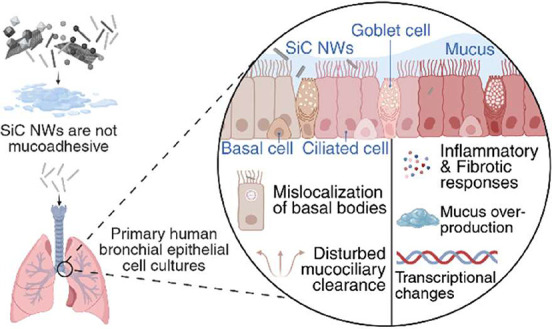

## Introduction

Advanced materials encompassing a diverse range of materials such as ceramics, polymers, two-dimensional (2D) materials, nanomaterials (NMs), and composites are characterized by significant improvements over conventional materials achieved through precise and controlled modifications.^1^ Silicon carbide (SiC) nanowires (NWs) are a notable example of silicon-based advanced materials, which first emerged in the 1970s, but are now more widely used in various applications.^2^ SiC NWs exhibit unique properties compared to their bulk counterparts, including nanoscale dimensions and exceptional mechanical, thermal, and electrical characteristics.^3,4^ Depending on the synthesis method, SiC NWs can be either crystalline or amorphous.^5,6^ Chemically, they are distinguished by lower polar surface groups, e.g., silanols.^7,8^ While the inhalation toxicity of crystalline and amorphous silica-based materials has been extensively studied, the toxicological profiles of advanced silica-based materials, such as SiC NWs, remain less explored. The association between various silica-based materials and respiratory diseases has been the subject of extensive research for decades.^9^ Critical structural properties contributing to their inhalation toxicity are the so-called nearly free silanols (NFS) or isolated silanol groups present on the surface of silica materials.^10,11^ Inhalation of crystalline silicon dioxide (cSiO_2_) dust can cause NLRP3 inflammasome-driven pulmonary inflammation leading to silicosis development.^12^ The pyrolytically processed amorphous SiO_2_ (aSiO_2_) (a/k fumed silica) NMs could also hold isolated silanol groups that could potentiate cell membrane damage by hydrogen-bonding interactions and enhanced generation of oxidative radicals, leading to cell death.^13^ In contrast, wet-produced SiO_2_ NMs are generally considered to have relatively low toxicity^13^ and are widely used for different industrial and consumer applications (e.g., paints, food packaging, drug delivery).^14–17^

The human respiratory system possesses self-clearing and innate defense mechanisms that protect distal lung tissue by removing inhaled particles and pathogens.^18,19^ Mucociliary clearance (MCC) is a key component of this self-clearing process, relying on factors such as proper cilia morphology, optimal cilia beating frequency (CBF), and adequate airway surface liquid levels (ASL).^20,21^ Respiratory mucus is a viscous polymer gel on the conducting airways, mainly constituted of water (97%) and mucin proteins (e.g., MUC5AB and MUC5AC).^22^ The mucin proteins facilitate building a polymer network (spacing of 400 nm) that could trap inhaled materials, including respiratory pathogens, and limit their exposure in the lung tissue and the rest of the body. Nonetheless, certain ultrafine particles, like respiratory pathogens, can bypass these protective mechanisms and rapidly accumulate in the distal airways as well as alveoli, potentially leading to pulmonary diseases.^21,23–25^ In the case of respiratory pathogens (e.g., SARS-CoV-2, Pseudomonas aeruginosa), mucociliary damage is the very first and essential step in the resulting mechanism of pathogenesis.^26,27^ Similarly, NMs (e.g., polymeric, carbon, SiO_2_, and metal oxides) have been shown to enter lung cells and cause toxicities, including fibrotic responses *in vitro* and *in vivo* upon long-term NM exposure.^28–32^ However, whether mucociliary damage could also be a key event in NM-mediated respiratory toxicity has not been well studied. Notably, NMs are often intentionally designed to bypass the mucociliary defense system for enhanced penetration to achieve the intended therapeutic applications. For example, PEGylation is one of the commonly used functionalization approaches to design NMs for lung applications since it renders them nonmucoadhesive and facilitates their entry into the lungs.^33,34^ In addition, NMs with bottlebrush PEG morphology have been shown to escape from the primary innate defense mechanism of the lungs.^35^

The pulmonary surfactant is another secreted fluid (type-II alveolar epithelial cells) in the lower respiratory tract that protects alveoli from collapsing by lowering the surface tension.^36^ Recent studies have shown that NM exposure can modulate physiological behavior and the function of lung surfactant. For example, Thai et al. incubated amine functionalized SiO_2_ (42 nm) and Al_2_O_3_ (40 nm) NPs with Curosurf (a porcine pulmonary surfactant) and showed strong interaction of both NPs with the lamellar bodies of pulmonary surfactant, which significantly affected surfactant flow properties. Authors also demonstrated that SiO_2_ NPs caused liquefaction of the pulmonary surfactant, whereas Al_2_O_3_ NPs induced solidification due to the particles’ (nonionic species) cross-linking ability with lung surfactant.^36^ In another study, Li et al. showed that pulmonary exposure to MWCNTs in mice affected lung surfactant formation by causing lamellar bodies dysfunction, which led to the elevation of surface tension and eventually lung fibrosis.^37^

The present study was designed to address the lack of understanding of the fate of inhaled SiC NWs in pulmonary mucosa, with a particular focus on mucociliary clearance, mucus penetration, ciliary damage, and subsequent downstream effects at cellular and molecular levels. Materials with well-studied respiratory toxicities (pyrolytic silica NPs, crystalline silica, MWCNTs, and graphene nanosheets) and new advanced NMs, SiC NWs, were included in the study to establish the role of mucociliary defense in the underlying toxicity mechanism. The cell line-based *in vitro* models are unsuitable for studying mucociliary functions, as they fail to fully recapitulate respiratory cilia, and do not secrete mucus that mimics physiological conditions,^38^ and there are also analytical difficulties in studying ciliary functions *in vivo* in real-time. Therefore, we used primary human bronchial epithelial (pHBE) cell cultures that were reconstituted using primary cells from a healthy human donor and cultured on a porous membrane under air–liquid interface (ALI) conditions. pHBE cell cultures retain an intact epithelial barrier and consist of the major cell types (ciliated cells, mucus-secreting goblet cells, and basal cells) from the bronchial regions (SI-Figure S1). These cultures fully recapitulate the necessary bronchial airway physiology (e.g., mucus secretion,^39,40^ motile cilia, and conducting microenvironment), thereby allowing extensive analysis of NM effects on mucociliary defense mechanisms. As illustrated in Scheme 1, we first investigated silicon- and carbon-based NM interactions with airway mucus and their potential transformation in the mucus-enriched acellular environment, recapitulating the upper airways. Next, the cell cultures were repeatedly exposed to NMs (one exposure/day) for 4 days under ALI conditions. To keep the realistic exposure dose limit of NMs for *in vitro* study as suggested elsewhere,^41,42^ the lower dose exposure was limited between 1–10 μg/cm^2^, while a higher dose (50 μg/cm^2^) was included only for dose–response analysis. Following exposure, the ciliary interactions of NMs, morphological changes, mucociliary function, NMs uptake, and their downstream effects in pHBE cells were studied. The results revealed a potential risk associated with SiC NWs exposure, including the ability to bypass mucociliary defense mechanisms, induce ciliary dysfunction, and trigger a fibrotic response. Our results further establish mucus penetration and mucociliary dysfunction as critical upstream key events in the toxicity mechanisms underlying SiC NWs-induced lung tissue damage.

**Scheme 1:**
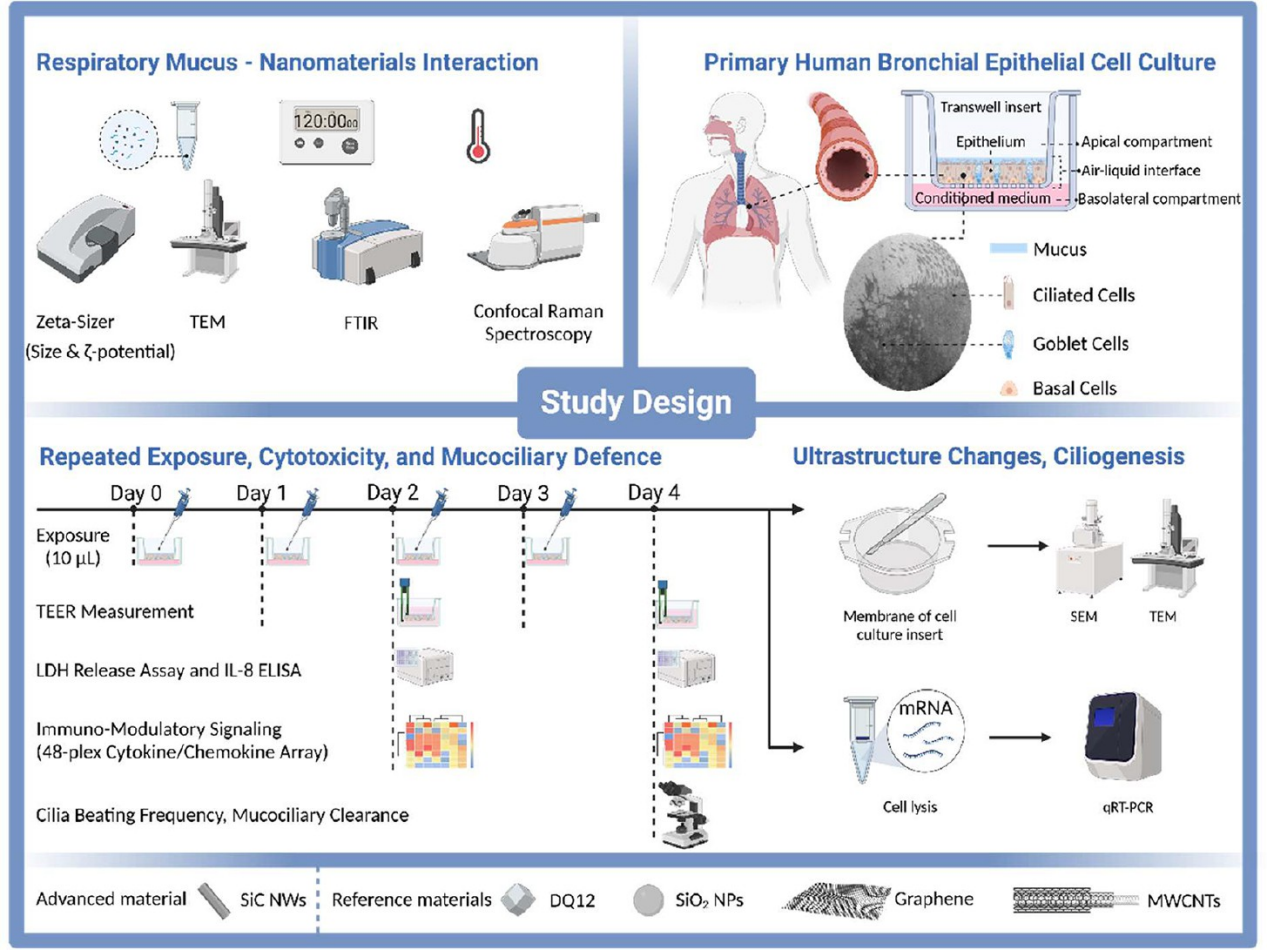
Study Design and Interactions of NMs (SiC NWs, SiO_2_ NPs, Graphene nanosheets, MWCNTs) and Crystalline Silica (Quartz DQ12, Reference Particle) with Respiratory Mucus Followed by Characterization Using a Range of Biophysical Analytical Approaches^a^

a Next, repeated exposure of NMs and quartz DQ12 at 1, 5, 10, and 50 μg/cm^2^ was conducted in pHBE cell culture for four consecutive days (applying 10 μL of particle suspension to the apical surface once per day). The effects on transepithelial electrical resistance (TEER, reflecting epithelial-barrier integrity), cell membrane damage (LDH release), and cytokine-chemokine secretion into the conditioned medium (basolateral) were measured on day 2 and day 4. However, effects on cilia, mucociliary functions, particle uptake, and transcriptional changes were evaluated after 4 days of exposure. The scheme was created with BioRender.com

## Results and Discussion

The physicochemical properties of the SiC nanowires (NWs), SiO_2_ NPs, graphene nanosheets, MWCNTs, and silica quartz (DQ12, reference material) are summarized in SI-Table S1. All the particles were verified for endotoxin contamination using the limulus amebocyte lysate (LAL) test and were endotoxin-free (<0.5 EU/mL, the threshold limit set by the United States Food and Drug Administration) at the highest analyzed concentration (50 μg/cm^2^) used for cell culture experiments.^43,44^

### SiC NWs Are Not Mucoadhesive and Penetrate through the Airway-Mucus Barrier

The mucus lining in the airway epithelium (secreted from goblet cells) is the first line of defense against inhaled NMs. It protects the lungs by trapping the NMs in the mucus gel and facilitating their subsequent clearance. Most NMs, once administered on the airway lumen, adhere to the airway mucus (known as mucoadhesive NMs) and undergo rapid physiological clearance from the lungs.^45^ On the contrary, NMs that do not attach to the airway mucus and are below 400 nm in size (smaller than the size of the mucus mesh spacings) can readily translocate through the mucus gel and trigger long-lasting effects within the bronchial epithelium.^46^ Taking this into consideration, we sought to clarify whether NMs could interact with airway mucus and then investigate how these interactions might lead to potential changes in the NM behavior. To study this, NMs were mixed with airway mucus collected from pHBE cell cultures and incubated for 2 h at 37 °C (Figure 1a). Following incubation, NMs were pelleted and used for further analysis in parallel with pristine NMs or airway mucus alone. Negative-staining transmission electron microscopy (TEM) was used to visualize the interaction between the NMs and airway mucus. As shown in Figure 1b, c, stained mucus components, predominantly proteins, were clearly visible on SiO_2_ NPs, MWCNTs, and graphene nanosheets (red arrows). However, partial to no interaction of mucus components was evident for silica quartz (DQ12) and SiC NWs, respectively.

**Figure 1:**
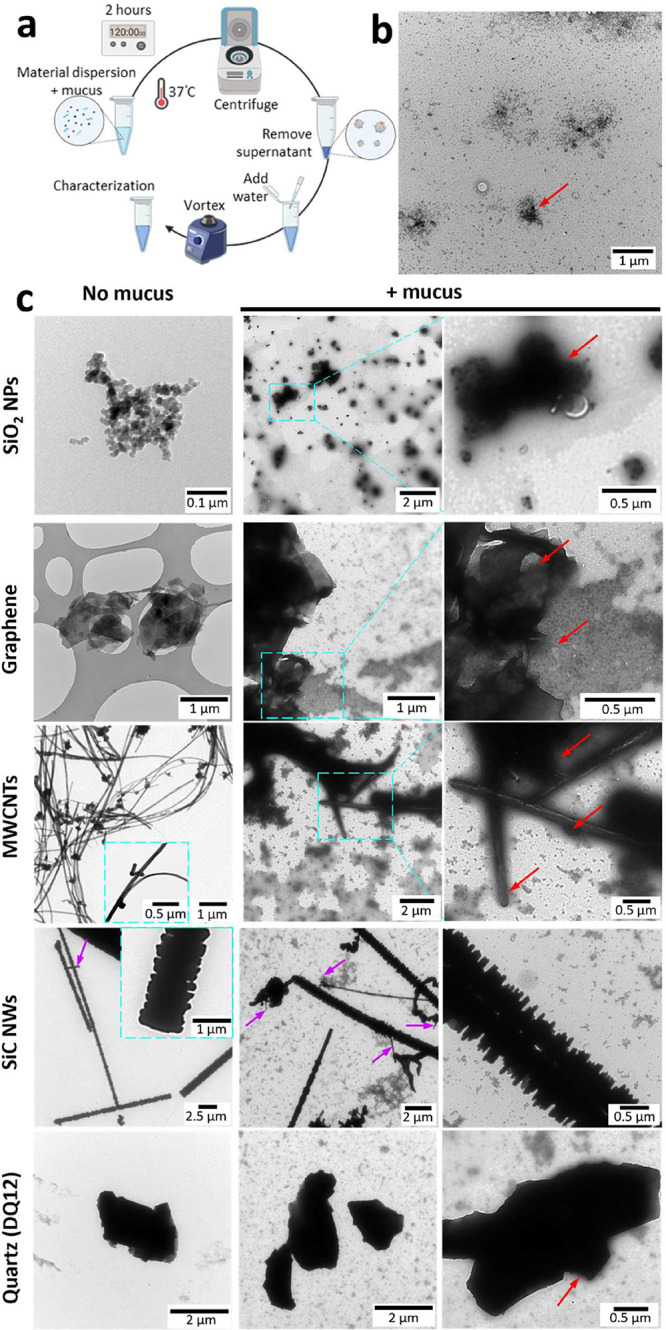
Negative-staining transmission electron microscopy (TEM) images show the surface interactions of SiO_2_ NPs, graphene, MWCNTs, SiC NWs and silica quartz (DQ12) with airway bronchial mucus components. (a) Materials were first dispersed in Milli-Q water and then mixed with airway mucus (collected from primary bronchial epithelial cells) or the same amount of water (negative control) and incubated for 2 h at 37 °C followed by removal of unbound mucus through precipitation of the particles with high-speed centrifugation. The settled particles were gently resuspended in Milli-Q water and used for characterization. (b, c) Negative-staining TEM images of mucus (b) and material dispersion before and after incubation with mucus (c). Red arrows indicate mucus components interacting with the materials. The mucus components, predominantly proteins, were stained for visualization and lead to dark stain accumulation around the materials in case of mucus interaction. Purple arrows indicate the presence of SiC nanowires with a small size and short length. Figure 1a was created with BioRender.com.

The surface reactivity of the NMs plays a significant role in their biological interaction and subsequent effects in cells, including biopersistence. The observed binding of mucus components to certain NMs may potentially be driven by electrostatic forces, since the NM surface chemistry has been shown to play a fundamental role in mediating interactions with biomolecules both extra- and intracellular.^47–49^ Indeed, a significant change in the surface ζ potential of the SiO_2_ NPs, MWCNTs, and graphene nanosheets was found after interactions with mucus compared to pristine NMs or mucus alone (Figure 2a). However, no obvious difference in surface ζ potential was measured for SiC NWs or quartz DQ12, in accordance with the visual observations by TEM (Figure 2a). Next, we examined whether interactions of NMs with mucus could lead to their agglomeration, which may impact NMs transport in the airway and eventually cellular entry. As shown in Figure 2b, the average hydrodynamic size of SiC NWs and quartz DQ12 was not changed after incubation with the airway mucus. However, an increase in the hydrodynamic size of graphene and SiO_2_ NPs was observed following 2 h of incubation with bronchial mucus compared to the size in Milli-Q water (without mucus) (Figure 2b), which could indicate particle agglomeration in the presence of mucus components. Interestingly, the hydrodynamic size of the MWCNTs was reduced after interaction with mucus (Figure 2b). This could potentially be explained by the formation of a biocorona (mainly from mucin proteins) on the particle surface, reducing the formation of large agglomerates or aggregates in the suspension, and thereby improving particle dispersion. In this context, previous studies have demonstrated that biocorona formation on certain NMs, including MWCNTs, can improve their dispersion in biological media.^50,51^ In fact, a low concentration of albumin protein (0.05–0.1% in water) is widely used to prepare a stable dispersion of MWCNTs for *in vitro* studies.^52,53^

**Figure 2:**
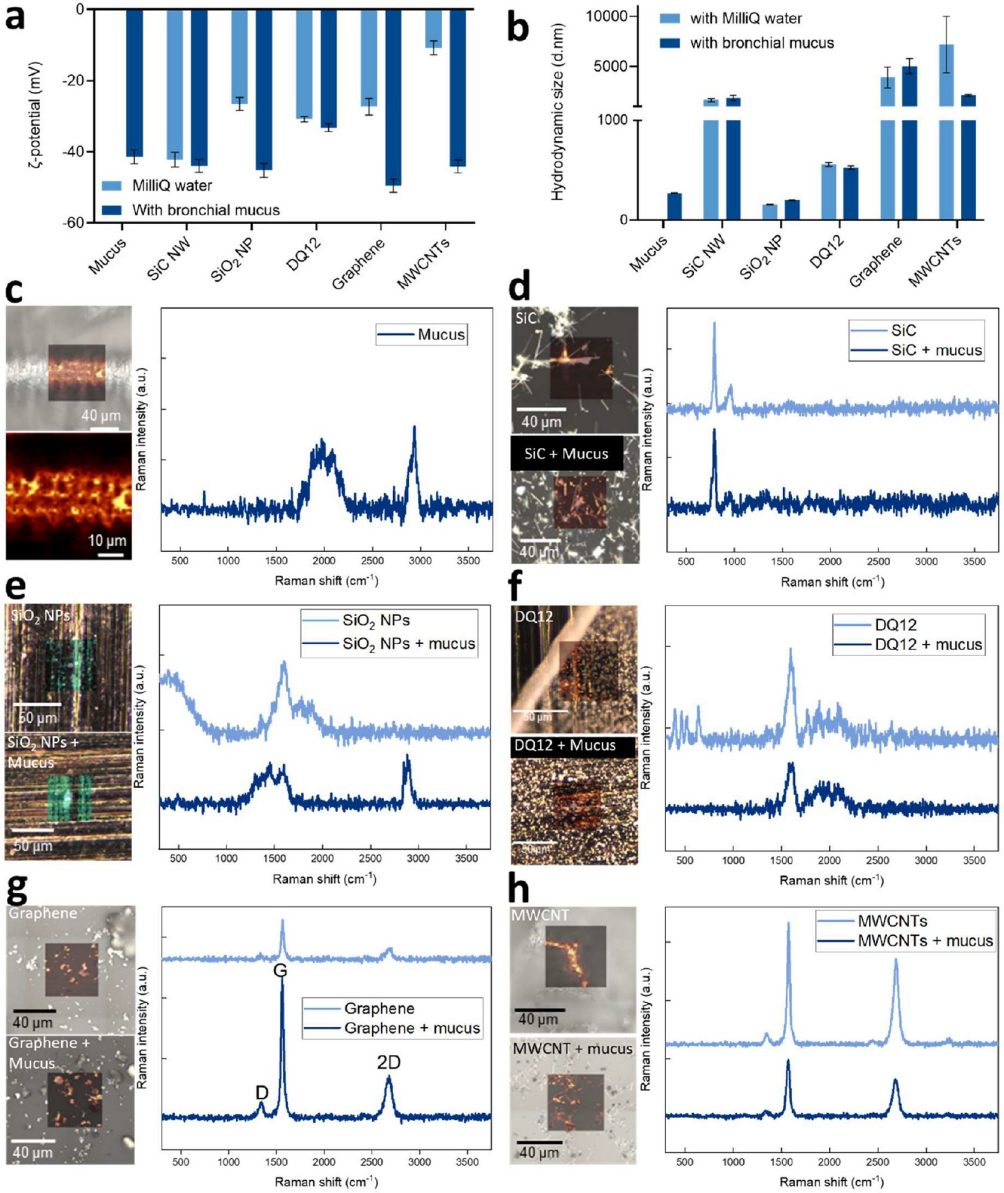
Changes in materials (SiO_2_ NPs, graphene, MWCNTs, SiC NWs and quartz DQ12) physicochemical properties after interaction with airway mucus. (a, b) Surface ζ potential (a) and hydrodynamic size (b) of the materials before and after 2 h of incubation with airway mucus. Data presented as mean ± SD from repeated measurements (*n* = 15). (c–h) Raman microscopy images and corresponding spectra from large-area scans (indicating scan area and observed signal from individual material and/or mucus): (c) mucus alone, (d) SiC NWs, (e) SiO_2_ NPs, (f) DQ12, (g) graphene, and (h) MWCNTs. a.u.: arbitrary units.

The presence or absence of mucus components on NMs and quartz DQ12 was further validated using Raman microscopy by large-area mapping of the NMs before and after incubation with airway mucus. In this analysis, airway mucus (without NMs) exhibited a net-like structure and revealed characteristic peaks at 2000 and 2936 cm^–1^ (SI Figure S2a). As provided in Figure 2c–h, the overlay images from large-area mapping and their corresponding spectra indicate the presence of mucus components on SiO_2_ NPs. However, no mucus fingerprints were observed for SiC NWs, quartz DQ12, and carbon-based materials (graphene and MWCNTs) before or after incubation with mucus, as revealed by the absence of a peak around 2936 cm^–1^. The absence of a mucus signature in the Raman spectra of carbon NMs could be because of relatively less mucus binding than SiO_2_ NPs, which is below the detection limit of the instrument. The spatial resolution limit of Raman microscopy is generally considered to be <1 μm, while the confocal resolution is <2 μm.^54^ Overall, this makes it challenging to analyze individual biomolecules in the mucus (size range of proteins: 10 to 300 nm).^55^ However, a cluster of proteins with optimal thickness can still be successfully analyzed using Raman microscopy, and such experimental approaches have been widely used for studying biomolecular corona formation on the NMs.^56,57^ It is also worth noting that in the case of graphene and MWCNTs, different acquisition times had to be applied due to signal saturation from the carbon NMs.

It is noteworthy that each NM exhibits distinct interactions with mucus, as demonstrated by TEM, dynamic light scattering (DLS), and Raman spectroscopy. Specifically, the adsorption pattern follows the order MWCNTs, graphene, SiO_2_ NPs ≫ quartz DQ12 > SiC NWs. However, this pattern does not appear to correlate with the size, shape (0D, 1D, and 2D materials), and surface area of the NMs. Furthermore, the supramolecular interactions between mucus components and NMs are primarily influenced by the binding affinity of the NMs’ surface organic groups to proteins, particularly the silanol groups in silicon-based NMs. The silanols or isolated silanol groups on silica nanoparticles or microparticle surfaces could play a crucial role in interactions with biomolecules and cells. Previous studies using *in vitro* cell cultures have demonstrated that an increase in silanol content on the silica surface could influence interactions with cells and biomolecules, leading to membrane lysis and cell death.^58–60^

Therefore, we performed Fourier-transform infrared spectroscopy (FTIR) analysis of SiO_2_ NPs, SiC NWs, and quartz DQ12 before and after incubation with mucus to unravel the potential role of silanol or isolated silanol groups in facilitating interactions with mucus components. The FTIR spectrum of mucus alone indicated the presence of aromatic and aliphatic groups (from the peptide residues) as well as amides, mainly representing an abundance of protein (mucins) and lipid molecules (SI-Figure S2b). The molecular structures of the silica particles suggest the presence of silanol groups on the SiO_2_ NPs and quartz DQ12, but not in SiC NWs (Figure 3a). FTIR results of pristine SiO_2_ NPs, SiC NWs, and quartz DQ12 confirmed the absence of silanol groups on SiC NWs and their presence in SiO_2_ NPs and quartz DQ12 (Figure 3b). SiO_2_ NP spectra in FTIR showed a shift in the vicinal (Si–OH) region of the spectrum (3720–3000 cm^–1^) following interactions with mucus, indicating the potential involvement of Si–OH groups in the interactions with mucus components (Figure 3b). Quartz DQ12 did not show a shift in the spectrum specific to silanol (Si–OH) groups but a decrease in transmittance, indicating physical adsorption of mucus components on the particle surface (Figure 3c). Interestingly, no such effects were evident in the FTIR spectra of SiC NWs following the incubation with mucus (Figure 3d).

**Figure 3:**
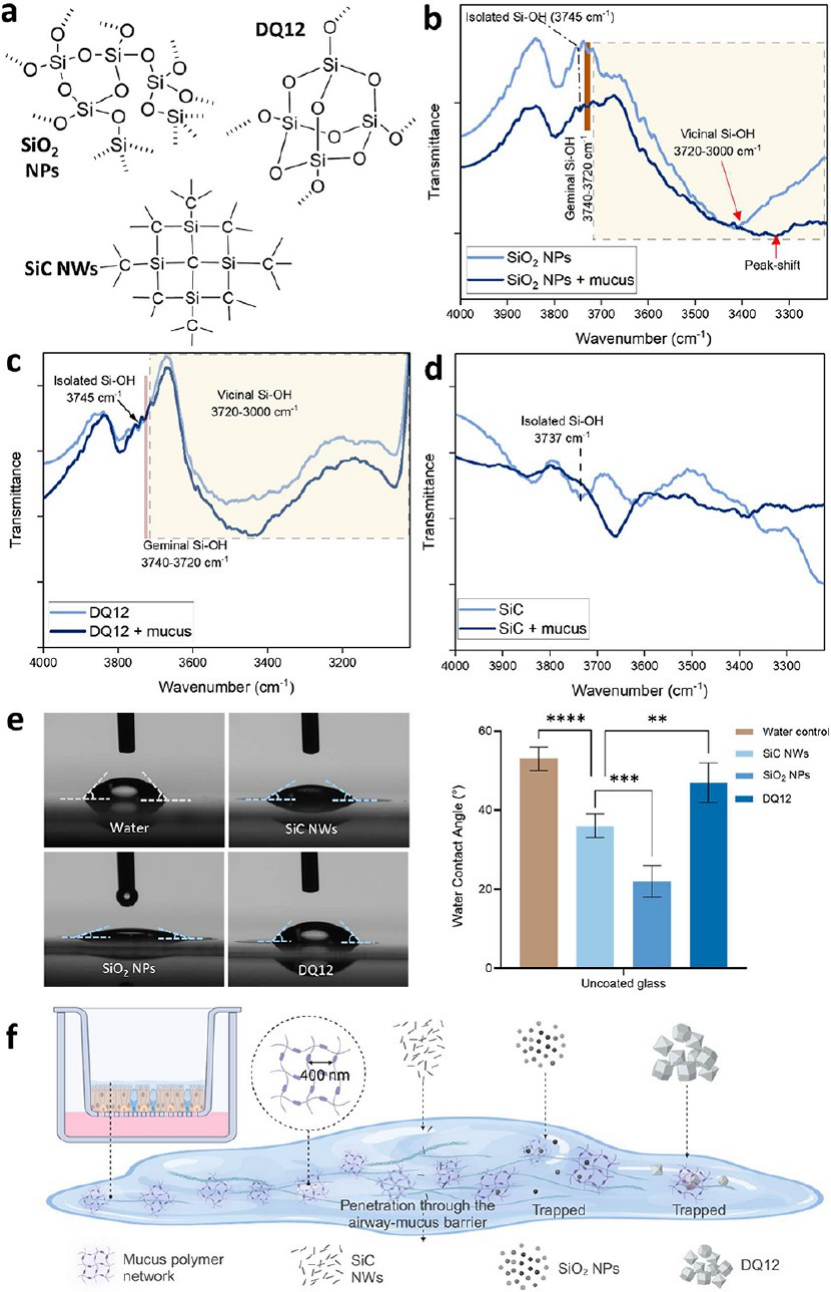
Fourier-transform infrared (FTIR) spectroscopy of SiO_2_ NPs, quartz DQ12 and SiC NWs revealed the potential role of surface silanols in mediating interactions with airway mucus. (a) Molecular structure of different silica particles showing the presence or absence of silanol (Si–O–H) groups. (b, c) FTIR spectra of SiO_2_ NPs (b), and quartz DQ12 (c) show the presence of different silanol moieties (geminal, vicinal, and isolated) in pristine materials, which were either shifted (in the case of SiO_2_ NPs) or changed in intensity after interaction with airway mucus. (d) FTIR spectra of SiC NWs before and after incubation with airway mucus indicate negligible silanol group density on the surface, suggesting the absence of strong interactions with airway mucus components. (e) Water contact angle measurement of indicated particles shows higher hydrophilicity of SiO_2_ NPs than the SiC NWs and quartz DQ12. Data presented as mean ± SD (*n* = 6). *p*-value was calculated by applying ordinary Two-way ANOVA and Dunnett’s multiple comparison test for *post hoc* analysis. **p < 0.01, ***p < 0.001, ****p < 0.0001 were considered statistically significant. *p* > 0.05 was considered not statistically significant (ns). (f) Illustration showing the interaction of SiO_2_ NPs, quartz DQ12 and SiC NWs with airway bronchial mucus. Mucus is a mixture of proteins, salts, lipids, and cell debris. This figure shows oversimplified mucus. Figure 3a was created with ChemDraw 23.0.1. Figure 3f was created with BioRender.com.

To further investigate the hydrophilicity of these NMs, we performed a water contact angle measurement, and the results are presented in Figure 3e. Among the three silica and silicon-based materials, SiO_2_ NPs exhibited significantly (*p* < 0.05) lower water contact angle than the quartz DQ12 and SiC NWs, indicating the higher hydrophilic behavior of SiO_2_ NPs than the other two materials. Consequently, SiO_2_ NPs are likely to interact with mucus more readily compared with SiC NWs and quartz DQ12. These results coincide well with the silanol density on the particles detected in the FTIR results (Figure 3b–d). The low hydrophilicity of quartz DQ12 (similar to SiC NWs) may be attributed to its microscale particle size and relatively low surface-to-volume ratio compared to nanomaterials.

Overall, our experimental model suggests that silanol groups play a critical role in facilitating the interactions of silica particles with mucus components, as illustrated in Figure 3f. The bronchial mucus is a highly hydrophilic gel due to its composition of mainly water (97%) and solids (3%), including mucins, nonmucin proteins, lipids, salts, and cell debris. On the other side, higher silanol density on the SiO_2_ NPs surface enhances their hydrophilicity, wettability, resulting in increased mucoadhesiveness in the bronchial epithelium.^22^ Taken together, the mucoadhesive particles will eventually be trapped in the bronchial mucus gel, and only very few particles may cross the periciliary layer and reach the cells. However, less or nonmucoadhesive particles (as shown here for SiC NWs) of sizes <400 nm will more readily penetrate the mucus gel and reach the bronchial epithelial cells. To prove this, we next investigated whether particles could cross the periciliary regions and internalize into bronchial epithelial cells.

Previous studies of NM-mucus interactions were mainly based on developing novel strategies to enhance NM penetration through mucus for therapeutic applications. For example, PEGylated NMs with bottlebrush morphology have been previously demonstrated to rapidly cross the mucus gel layer in the airways.^33,61^ In a similar context, Suk et al. showed that pretreatment with a mucolytic compound (N-acetyl cysteine (NAC) could further increase the mucus penetration of PEGylated NMs.^62^ In a study performed using pig pulmonary mucus, carboxyl functionalized polystyrene NPs smaller than 200 nm in size were able to freely diffuse through the mucus polymer network, whereas 500 nm particles were locally trapped in the mucus.^63^ Guo et al. investigated the effect of surface functionalization (using amine and carboxyl groups) of silica NPs on mucus penetration.^64^ Their results revealed that amine- and carboxyl-functionalization of silica NPs led to electrostatic interactions and hydrogen bonding with mucin proteins, which inhibited their diffusion through the mucus. In contrast, the authors also demonstrated that covering these silica NPs with high-density PEG molecules shields particle-mucin interactions and enables free diffusion across the mucus layer. Overall, while there is intense research on the development of nonmucoadhesive coating strategies for more effective NP-based drug delivery, there is a lack of data on the health consequences of nonmucoadhesive advanced materials after penetrating the protective mucus barrier.

### SiC NWs Damage the Ciliary Layer and Thereby Breach the Mucociliary Innate Immune Defense of Human Bronchial Epithelial Cells

The clearance of inhaled NMs from the upper respiratory airways is decisively dependent on their physical association with extracellular mucus. In the mucociliary clearance process, inhaled particles trapped in airway mucus encounter beating cilia and are cleared from the conducting airways. Nevertheless, NMs that can diffuse through the mucus gel or impair ciliary function may cause negative impacts on the upper airway due to higher uptake by the cells. In pathological analysis, impaired mucus production and/or loss of ciliary functions are also considered as one of the most common signs of airway diseases, since it can make individuals more susceptible to respiratory damage.^65,66^ The most evident examples in this context are respiratory pathogens, which first target respiratory cilia or ciliated epithelial cells. This disruption impairs mucociliary clearance, ultimately enabling the pathogens to penetrate deeper into the lungs.^26,27^ As discussed above, SiC NWs showed the least association with mucus components, which might imply their facilitated entry into the ciliary compartment and ultimately into the lung cells.

Therefore, we first investigated whether SiC NWs and other materials could interact with airway cilia and affect their functions. To this end, the pHBE cell cultures were repeatedly exposed to the materials for 4 days, and changes in ciliary morphology and functions were analyzed. As visible in Figure 4a, scanning electron microscopy (SEM) analysis revealed well-developed, long cilia with unidirectional orientation on the apical surface of untreated cell cultures (vehicle control). However, SiC NWs exposure (10 μg/cm^2^) triggered shorter cilia, a partial loss, and a disheveled orientation of motile cilia, revealing the nonciliated goblet cells (GCs, white dashed areas, Figure 4b) that are usually covered by the long cilia of healthy ciliated cells, and deciliated areas with cells containing shorter or flattened cilia (yellow dashed areas, Figure 4c). No changes in ciliary morphology were visible in pHBE cultures exposed to SiO_2_ NPs, quartz DQ12, graphene, or MWCNTs (SI-Figure S3a–d). We further showed that the loss of motile cilia from SiC NWs exposure resulted in a significant decrease in CBF at all tested concentrations (Figure 4d). To assess the MCC, the movement of polystyrene beads (indicating clearance by cilia) added onto the apical pHBE surface was tracked following 4 days of exposure to SiC NWs or in control cell cultures. This is a well-established method to measure ciliary functions in *in vitro* cell cultures.^25^ MCC of the applied beads was recorded in real-time, revealing a reduced velocity of beads movement in pHBE cultures exposed to SiC NWs (10 and 50 μg/cm^2^) compared to the untreated control (Figure 4e, SI Videos 1, 2, and 3). While a unidirectional movement of polystyrene beads was clearly visible in control cell cultures (SI Video 1), SiC NWs-exposed cultures either showed a slow and nonunidirectional movement of beads at low dose exposure (10 μg/cm^2^) (SI Video 2) or a nearly complete loss in movement at a higher exposure dose (50 μg/cm^2^) (SI Video 3), consistent with the observed effects in cilia orientation and morphology in SEM. MWCNTs-exposed cell cultures also exhibited significantly reduced CBF at 10 and 50 μg/cm^2^ (SI Figure S4a), however, no significant (*p* > 0.05) effects were observed in MCC function of the pHBE cell cultures (SI-Figure S4e). The graphene nanosheets, SiO_2_ NPs, and quartz DQ12 exposure did not cause significant (*p* > 0.05) effects in either the CBF (SI-Figure S4b–d) or MCC functions of pHBE cell cultures (SI Figure S4f–h).

**Figure 4:**
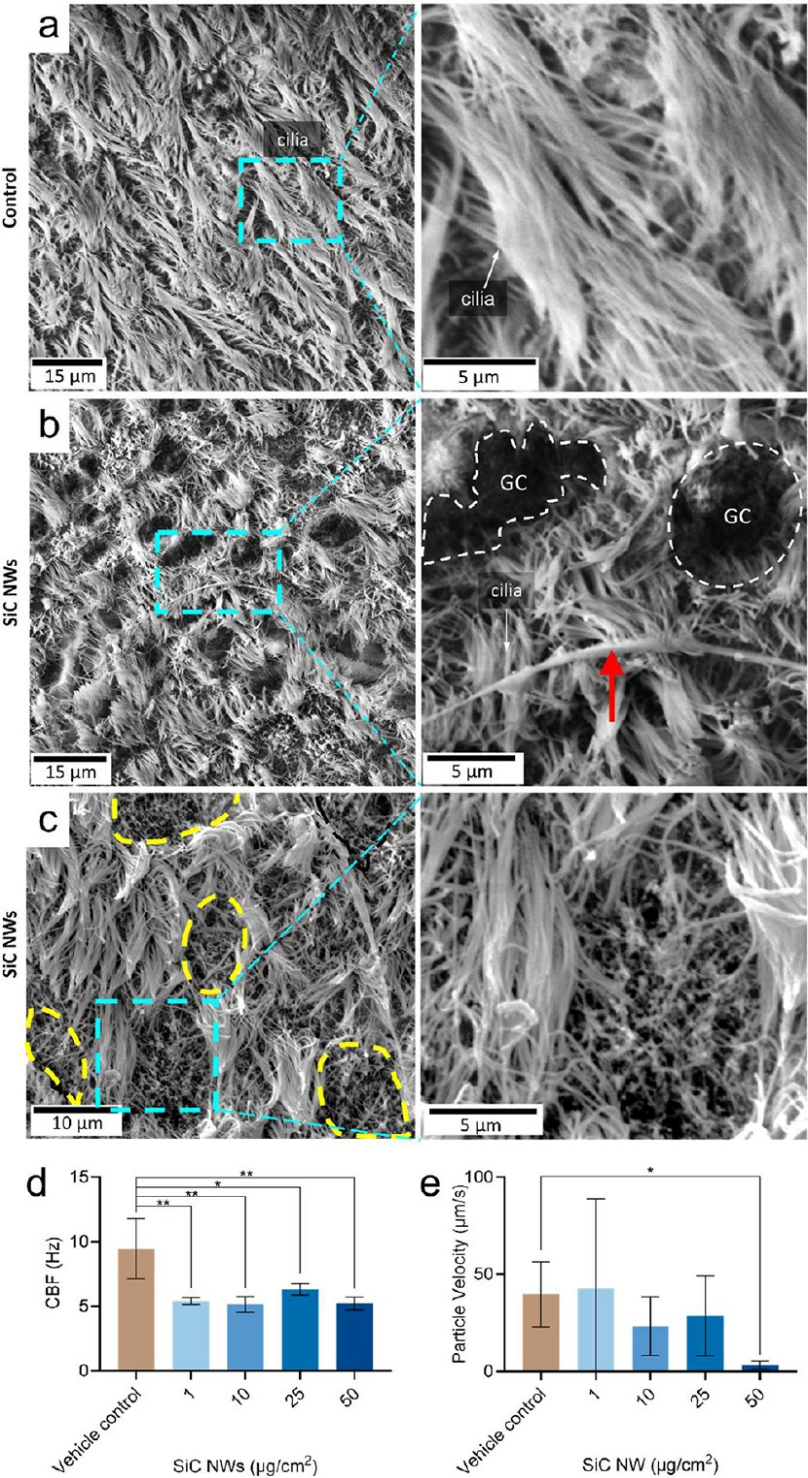
SiC NWs triggered ciliary damage and affected the mucociliary clearance function of pHBE cell cultures after 4 days of repeated exposure. Scanning electron microscopy (SEM) showing ciliary morphology in (a) vehicle control (0.05% BSA in 0.9% NaCl solution) and (b, c) after exposure to SiC NWs (10 μg/cm^2^). (a) Well-developed, long cilia with unidirectional orientation are visible on the apical surface of untreated cell cultures in SEM (b) SiC NWs (red arrow) induced a disheveled orientation, loss of cilia and shorter cilia, and shortening of cilia, revealing the nonciliated goblet cells (GC, white dashed areas) underneath that are usually covered by the long cilia of healthy ciliated cells. (c) Deciliated areas (yellow dashed areas) with short, flattened cilia were also visible, indicating a loss or reduction of cilia on ciliated cells. (d) SiC NWs significantly reduced CBF at indicated doses. (e) Mucociliary clearance function (measured as the velocity of microbead clearance) was reduced at the highest exposure dose of 50 μg/cm^2^ SiC NWs. Data presented as mean ± SD (*n* = 3). The *p*-value was calculated by applying ordinary One-way ANOVA and Dunnett’s multiple comparison test for post hoc analysis. *p < 0.05, **p < 0.01, ***p < 0.001, ****p < 0.0001 were considered statistically significant. *p* > 0.05 was considered as not statistically significant (ns). Please see SI-Figure S4 for CBF and MCC results of pHBE cell cultures exposed to SiO_2_ NPs, MWCNTs, quartz DQ12, and graphene. Note also the SI-videos (SI Videos 1, 2, and 3) visualizing the effects of SiC NWs (10 and 50 μg/cm^2^) on mucociliary function in real-time.

Due to the requirement for advanced lung culture models with functional beating cilia and physiological mucus secretion (e.g., reconstituted pHBE 3D lung cultures), only a few previous studies have so far investigated the mucociliary effects of NMs, mainly for MWCNTs in acute and subchronic exposure scenarios. Using pHBE cell cultures from healthy and asthmatic donors, Chartorea et al. could show that repeated exposure to MWCNTs at an occupationally relevant dose (10 μg/cm^2^ for 5 weeks of repeated exposures/5 days per week) enhanced CBF in both healthy and asthmatic cell cultures, whereas MCC was only affected in the latter.^67^ No other cytotoxic or phenotypic effects were reported in the study. In contrast, Beyeler et al. investigated acute exposure assessment (24 h) of MWCNTs (including asbestos and quartz DQ12 as positive control materials) in pHBE cell cultures from healthy and COPD patients, and results showed no effects on CBF for any of the conditions analyzed for MWCNTs and positive controls.^68^ Moreover, MWCNTs were taken up by epithelial cells (mainly goblet cells) but were unable to cause any effects on either cell viability or gene expression for oxidative and inflammatory markers when compared to quartz DQ12.

Several *in vivo* studies have evidenced that inhalation exposure to certain NMs, including silica dust and high aspect ratio materials (e.g., carbon and boron-based nanotubes) can trigger mucus hypersecretion and bronchial thickening, a leading cause of certain lung diseases (e.g., asthma, COPD).^28^ ,^69^ ,^70^ Carbon NPs of 14 nm diameter have been shown to enhance OVA-induced allergic inflammation and mucus hypersecretion.^71^ Similarly, CuO NP exposure in asthmatic mice has been shown to induce mucus hypersecretion, leading to aggravation of the disease.^72^ TiO_2_ NPs (size < 75 nm) exposure in human bronchial epithelial cells (ChaGo-K1) has been demonstrated to enhance mucin secretion via the calcium-mediated signaling pathway.^73^ Yu et al. conducted 84 days of pulmonary exposure analysis of silica microparticles in mice, and their results revealed impaired mucociliary defense because of mucus hypersecretion, decreased MUC5B expression, and ultrastructural defects in airway cilia.^31^ Finally, the authors also suggested that effects on cilia could be a key upstream event in the development of silica-mediated respiratory diseases.

Overall, our results show that SiC NWs could bypass mucociliary defense and cause structural and functional defects in airway cilia. Mechanistically, the observed effects on ciliary function could result either from physical damage to the cilia caused by SiC NWs or from intracellular mechanisms triggered upon particle exposure or uptake. Thus, we next studied whether SiC NWs can enter cells and cause cytotoxicity or modulate cellular processes related to ciliogenesis and mucociliary functions.

### SiC NWs Trigger Ultrastructural Changes and Induce Expression of the Ciliogenesis Regulator *FOXJ1* in Human Bronchial Epithelial Cells

TEM analysis of pHBE cell cultures was performed to investigate potential cellular uptake and ultrastructural changes following exposure to SiC NWs (Figure 5) and other materials (SiO_2_ NPs, quartz DQ12, Graphene nanosheets, and MWCNTs, SI-Figure S5) compared to the vehicle control. Occasionally, structures resembling SiC NWs were observed in the cytoplasm of exposed ciliated cells (Figure 5b-ii, SI-Figure S6, red arrows), localized within membrane-bound endosomes that appeared to be fusing together from their spatial proximity (SI-Figure S6, yellow arrows). Elongated structures resembling SiC NWs were also found in the extracellular ciliary region (Figure 5b-iii), whereas no such structures were found in untreated pHBE cells (vehicle control; Figure 5a). Indeed, polarized epithelial cells are capable of endocytosis^74^ and ciliated epithelial cells, being a subtype of polarized epithelial cells, also exhibit endocytic activity.^75^ Abariute et al. showed that alveolar epithelial adenocarcinoma cells can internalize nanowires with a length of around 5 μm through processes requiring dynamin and actin polymerization, suggesting that phagocytosis and macropinocytosis were involved.^76^ In the present study, the length of SiC nanowires found within the cell was also in the size range of 0.5–5 μm. Therefore, it is possible that SiC NWs entered the ciliated cells via endocytosis. Moreover, particles resembling SiO_2_ NPs, quartz DQ12, graphene nanosheets, and MWCNTs were also rarely observed inside epithelial cells or in the extracellular ciliary region (SI-Figure S5). These particles may have entered the cells from the thinner mucus regions due to the uneven distribution of mucus across the surface of the pHBE cell cultures. However, it is noteworthy that these observations do not provide a statistical quantification of particle uptake since TEM imaging is rather a qualitative approach performed only on several 2D sections at different *z*-axis depths of the sample. As the NMs assessed here have hardly detectable elemental signals (also given low cellular uptake) over the background of the cells and TEM/SEM sample holder (grids, wafers, grid holder) in energy dispersive X-ray spectroscopy (EDX), the identity of the particles observed inside the cells or in the extracellular ciliary region could only be evaluated from their morphology compared to the native particles or particles with mucus as in Figure 1.

**Figure 5:**
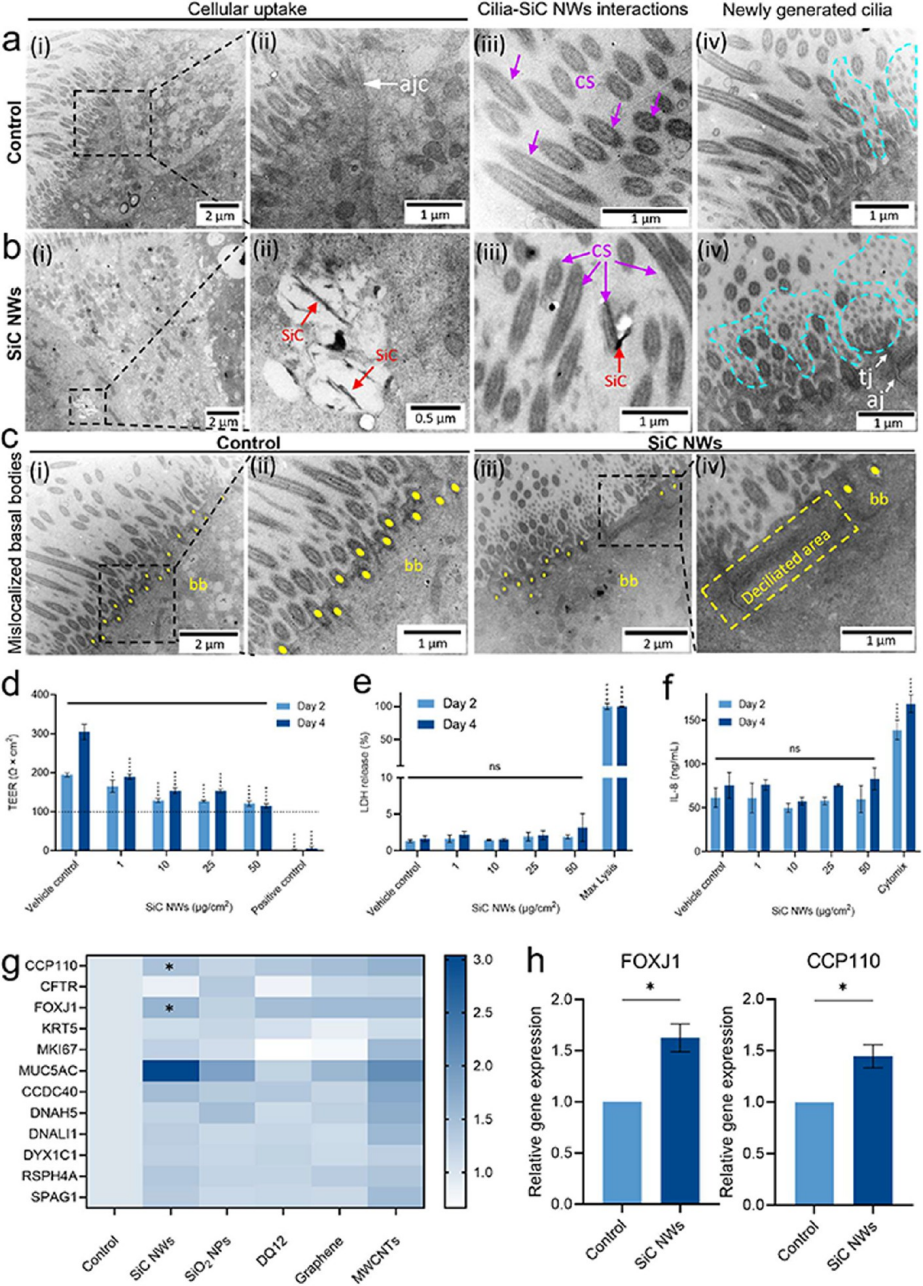
SiC NWs are found in the extracellular ciliary region and cytoplasm of epithelial cells and trigger ultrastructural and transcriptional changes related to cilia function. TEM images of pHBE cell cultures showing particle uptake and ultrastructural changes in cilia after 4 days of repeated exposure: (a) vehicle control (0.05% BSA in 0.9% NaCl solution) and (b) SiC NWs (10 μg/cm^2^). (b) Red arrows show the presence of SiC NWs in endosomes of ciliated cells and in the extracellular ciliary area. The highlighted region of interest (black dashed square) in (a–c) is presented as a zoomed image on the right. Any effects on intercellular apical junctional complex (apical junctional complex–j, tight junctions–tj, adherens junctions–aj, white arrows) and ciliary morphology (cilia section–cs, purple arrows; cross sections of newly generated cilia–cyan dashed areas; basal body–bb, yellow dots; diciliated area–yellow dashed area) are shown in representative TEM images. TEM images showing the uptake of SiO_2_ NPs, quartz DQ12, graphene, and MWCNTs are presented in SI-Figure S5. Refer to SI-Figure S6 for additional TEM images highlighting the presence of SiC NWs in endosomes and intact intercellular apical junctional complex. (d) Transepithelial-electrical resistance (TEER) of pHBE cell cultures was reduced in a dose-dependent manner following 2 and 4 days of exposure to SiC NWs. The dashed line in the graph indicates the threshold TEER value for an intact epithelial barrier. (e) No significant LDH release from pHBE cell cultures was observed after days 2 and 4 of repeated exposure to SiC NWs at any of the assessed doses with respect to the vehicle control (0.05% BSA in 0.9% NaCl solution). (f) No increase in IL-8 secretion was observed at any assessed dose of SiC NWs with respect to the vehicle control (0.05% BSA in a 0.9% NaCl solution). See SI-Figure S6 for TEER and LDH release results and SI-Figure S7 for IL-8 results of pHBE cell cultures exposed to SiO_2_ NPs, quartz DQ12, graphene, and MWCNTs. Data presented in parts (d–f) are mean ± SD from three independent cell cultures (*n* = 3). The statistical significance (*p*-value) between vehicle control and SiC NWs was calculated by applying Two-way ANOVA and Dunnett’s multiple comparison post hoc test. *p < 0.05, **p < 0.01, ***p < 0.001, ****p < 0.0001 were considered statistically significant. *p* > 0.05 was considered as not statistically significant (ns). (g) Heat map showing fold change in expression of mRNA transcripts for the indicated genes (related to ciliary structure, ciliogenesis, ciliary functions, and mucus production) after 4 days of repeated exposure to SiC NWs, SiO_2_ NPs, MWCNTs, quartz DQ12, and graphene at 10 μg/cm^2^. *p*-value was calculated by applying the unpaired Welch’s *t* test. *p < 0.05 was considered statistically significant with respect to control. (h) Bar plots showing a statistically significant increase in transcripts related to ciliogenesis after SiC NWs exposure. Data is presented as mean ± SD and the statistical significance (*p*-value) between vehicle control and SiC NWs was calculated by applying unpaired Welch’s *t* test. *p < 0.05 is considered statistically significant.

The ultrastructure analysis of untreated pHBE cell cultures with TEM revealed a typical arrangement of ciliated cells and goblet epithelial cells on the apical side with intact intercellular apical junctional complex (ajc, Figure 5a-ii, white arrow)^77,78^ Moreover, the ciliated cells in untreated pHBE cultures displayed a characteristic ultrastructure with the long cilia anchored in the cytoplasm via basal bodies aligned in a zigzag manner (bb, Figure 5c-i-ii, yellow dots). Cross sections of the cilia (cs, Figure 5a-iii,b-iii, purple arrows) were further visible with two central microtubules surrounded by pairs of dynein arms.^65^ In contrast, the ciliated cells in the SiC NWs-exposed pHBE cultures showed slight ultrastructural differences compared to the untreated ciliated cells (Figure 5b-iv,c-iii,iv): a higher number of newly generated cilia (smaller in length and thinner in diameter) were evident in SiC NW-exposed cells (Figure 5b-iv, cyan dashed areas) as compared to the respective control (Figure 5a-iv). A trend toward a more uneven distribution of basal bodies along the cell membrane was observed, with a reduced zigzag pattern (bb, Figure 5c-iii-iv, yellow dots), visible gaps (deciliated area, Figure 5c-iv, highlighted with yellow dashed lines), and mislocalization from the cell membrane, further extending into the cytoplasm (bb, Figure 5c-iv, yellow dots). These observed ultrastructure changes in cilia and basal bodies in pHBE cell cultures after SiC NWs exposure further support the findings of reduced cilia beating and mucociliary clearance functions, as shown in Figure 4d,e.

In addition to the ciliary malfunctioning, NMs exposure could also affect the epithelial barrier function of pHBE cell cultures either by disrupting intercellular apical junctional complex (tight junctions, adherens junctions, and desmosomes)^78^ and/or modulating cell membrane ion channels. To examine the effect on epithelial barrier functions of pHBE cell cultures, we determined changes in transepithelial electrical resistance (TEER) after days 2 and 4 of exposure to SiC NWs and other materials. As shown in Figure 5d, a dose-dependent (1–50 μg/cm^2^) and statistically significant (*p* < 0.05) decrease in TEER was observed after exposure to SiC NWs at both time points. Interestingly, other tested materials also triggered a drop in TEER values (Figure S7a–d). However, it is important to note that the decrease in TEER value was still above the threshold TEER value of 100 Ω·cm^2^ based on empirical observation for pHBE cell cultures, which implies that the epithelial barrier was not severely damaged. In our TEM analysis, we did not observe any significant alterations in the tight junctions (tj, Figure 5b-iv, white arrow) or adherens junctions (aj, Figure 5b-iv, white arrow) of cells exposed to the NMs, including SiC NWs (Figure 5a,b). Therefore, the observed reduction in TEER values could be a consequent effect of ion channels operating on the surface of the epithelial cell layer. However, future studies are warranted to establish the role of ion channels. Given the observed effects on epithelial-barrier integrity of pHBE cell cultures after exposure to NMs and quartz DQ12, we next assessed if the cell viability of pHBE cultures was affected. All studied materials, including SiC NWs, did not induce a significant (*p* > 0.05) increase in LDH release (indicating cell membrane rupture) from cells at any of the analyzed concentrations on either day 2 or day 4, reflecting no effect on the cell viability (Figure 5e and SI-Figure S7e–h).

We next examined interleukin IL-8 secretion from the cells exposed to SiC NWs and other materials, since IL-8 is a proinflammatory cytokine expressed by most epithelial cells, including those in the lungs, and is frequently used for initial screening of acute inflammatory response due to tissue damage.^79^ As shown in Figure 5f, IL-8 secretion was not significantly increased after exposure to SiC NWs on either day 2 or day 4 compared to the vehicle control. Similarly, none of the other materials (SiO_2_ NPs, quartz DQ12, MWCNTs, and graphene) induced the release of IL-8 after 2 and 4 days of repeated exposure (SI-Figure S8a–d).

Given the high aspect ratio of the SiC NWs (diameter 0.1–1 μm, length 10–50 μm, SI-Table S1), their cytotoxicity could also be governed by the long and rigid structure, similar to other high aspect ratio nanomaterials (HARNs), e.g., Mitsui7MWCNTs. For instance, previous studies have shown that long (diameter approximately 50 nm, length ≥ 10 μm) and rigid MWCNTs can penetrate through cell membranes and induce cellular injury characterized by increased inflammation, fibrotic changes, and granuloma formation.^80,81^ On the other hand, Fubini et al. demonstrated that chemical composition and surface state of the NMs also play a crucial role in the cytotoxicity of HARNs.^82^ Taking chemical properties into consideration, Si–C bonds present in SiC NWs are more polar than the C–C bonds in MWCNTs and therefore could undergo hydrolysis, leading to a low biopersistence of these materials.^83^ Although both NMs are HARNs, the long-term toxicity of SiC NWs may differ from that of the long and rigid MWCNTs.

To further understand if the largely observed effects on barrier integrity and mucociliary functions were accompanied by changes in gene expression levels, we performed mRNA expression analysis using quantitative RT-PCR for key genes (SI-Table S2) involved in regulating ciliogenesis, ciliary functions, and mucus production. As shown in Figure 5g,h, SiC NWs triggered a statistically significant (*p* < 0.05) increase in transcripts encoding the ciliogenesis regulators *FOXJ1* and *CCP110* (Figure 5h). *FOXJ1* is a well-known transcription factor that regulates ciliogenesis in lung epithelial cells.^84–86^ A noticeable upregulation of *MUC5AC,* expressed in goblet cells, was also observed (Figure 5g), suggesting an increased mucin production that may lead to mucus hypersecretion.^87^ This could be a response of the epithelial cell layer to hinder the entry of the NMs from the extracellular ciliary region into their cytoplasm via obstruction through mucus, since the ciliary clearing functions were impaired.^87^ In addition, a slight decrease (not statistically significant) in transcripts encoding cystic fibrosis transmembrane regulator (*CFTR*) was recorded, indicating potential effects on airway surface liquid exchange.^88^ The downregulation of *CFTR* expression could be a subsequent effect of mucus hypersecretion. On the other hand, upregulation of *FOXJ1* expression could be a compensatory effect to the damaged cilia after exposure to SiC NWs. SiO_2_ NPs, quartz DQ12, MWCNTs, and graphene nanosheets did not have a statistically significant (*p* > 0.05) effect on the transcripts related to ciliogenesis, ciliary functions, or mucus production (Figure 5g).

In a previous study, carbon NPs produced by the spark-ablation method and then exposed to bronchial lung epithelial cells (16HBE14o) have been shown to downregulate the expression of *CFTR*.^89^ McCarthy et al. (2011) have shown that polystyrene NPs (20 nm) with negatively charged surfaces can directly activate CFTR Cl^–^ channels in human submucosal lung cells (Calu-3) and baby hamster kidney cells, which led to an increase in short-circuit current.^90^

Ciliary dysfunction is a frequently observed phenomenon in individuals with a smoking history, which makes them prone to air pollution and respiratory infections.^91^ Therefore, the pronounced adverse effects of SiC NWs on ciliary structure (e.g., loss, mislocalization, and altered ultrastructure of cilia and basal bodies) and function (impaired CBF and MCC), could also facilitate the entry of other toxic materials or respiratory pathogens into the lung tissues and potentially lead to the development of respiratory diseases in the long term.

### SiC NWs Induce Inflammatory and Pro-Fibrotic Responses in Human Bronchial Epithelial Cells

Repeated exposure to quartz DQ12 and MWCNTs has previously been shown to promote pro-inflammatory reactions via the production of cytokines and chemokines in 3D bronchial lung epithelial cell cultures, which could sensitize the airways.^92,93^ The cytokine-chemokines secretion has also been demonstrated to affect mucociliary defense by promoting mucus hypersecretion and deposition in the airways.^94^ Therefore, we next investigated potential pro-inflammatory or pro-fibrotic cytokine/chemokine responses in pHBE cell cultures after exposure to SiC NWs and other materials (SiO_2_ NPs, quartz DQ12, graphene, and MWCNTs). To this end, multiplex cytokine array (48-plex) measurements were performed from the basolateral conditioned medium collected after day 2 and day 4 following exposure. Hierarchical cluster analysis of the cytokine-chemokine array results suggested that SiC NWs and quartz DQ12 exposures (10 μg/cm^2^) had strong effects on the secretion of pro-inflammatory and pro-fibrotic interleukin and chemokine factors after day 2 (SI-Figure S9) and day 4 (Figure 6a). CytoMix was used as a positive control and had the strongest effects on most of the cytokines-chemokines analyzed in the array after day 2 (SI-Figure S9) and day 4 (Figure 6a) postexposure. The other materials also induced moderate effects on cytokine-chemokine secretion after days 2 and 4 of exposure, as summarized in Table S3. SiC NWs results were further analyzed, and the cytokines/chemokines that were significantly (*p* < 0.05) affected with respect to vehicle control were plotted in two groups: pro-inflammatory (Figure 6b) and pro-fibrotic (Figure 6c).

**Figure 6:**
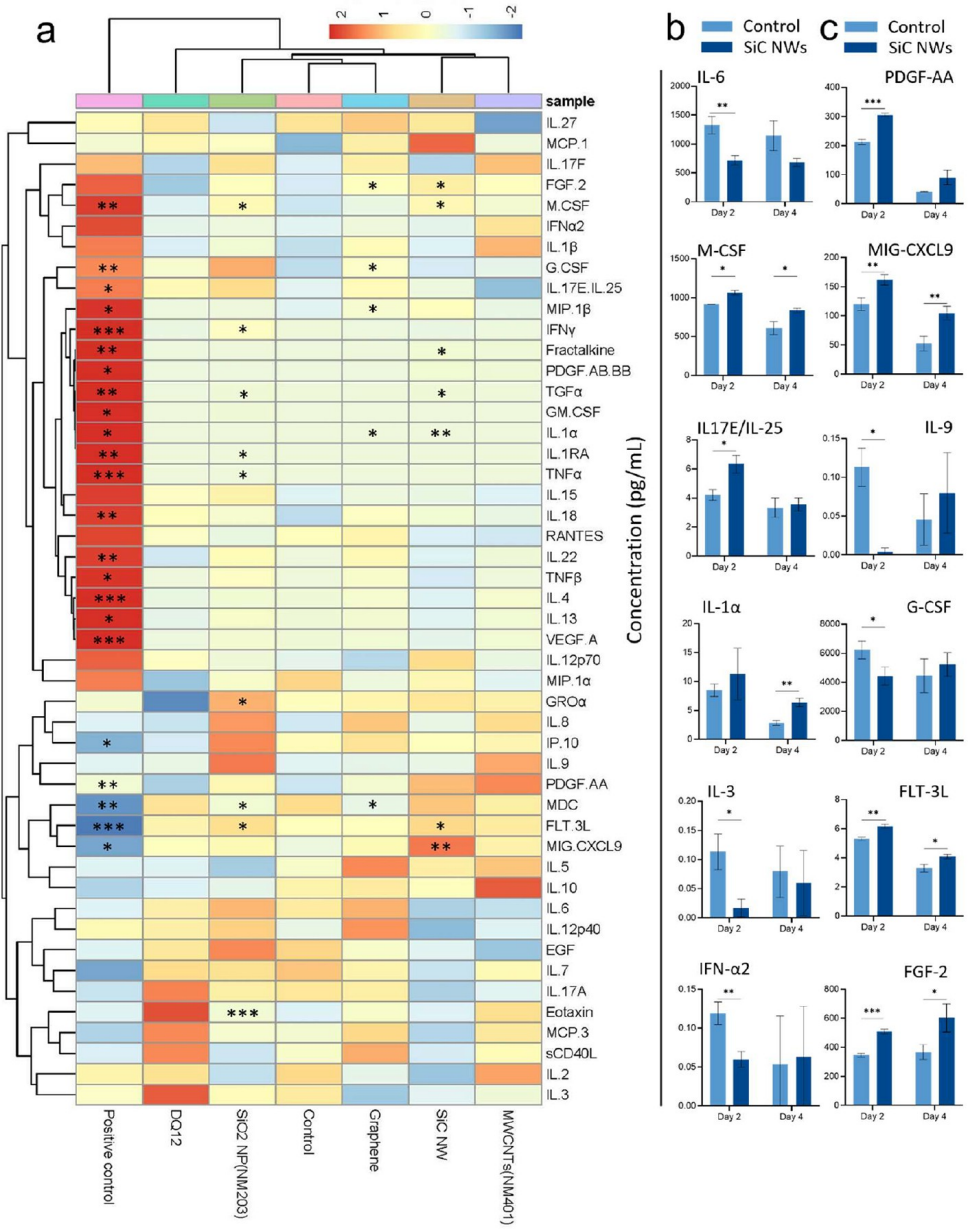
Cytokine-chemokine responses in pHBE cells after 2 and 4 days of exposure to SiC NWs, SiO_2_ NPs, MWCNTs, quartz DQ12, and graphene nanosheets. (a) Heat map with hierarchical clustering shows global cytokine-chemokine responses after 4 days of exposure to the indicated materials. 0.05% BSA in 0.9% NaCl solution was used as a negative control and CytoMix was used as a positive control. (b, c) Detailed graphs of selected cytokines from SiC NWs exposed pHBE with statistically significant effects on pro-inflammatory (b) and pro-fibrotic cytokine-chemokine factors. Data in (b, c) are presented as mean ± SD (*n* = 3). The *p*-value was calculated by applying the unpaired Welch’s *t* test. *p < 0.05, **p < 0.01, ***p < 0.001 were considered statistically significant with respect to the control. *p* > 0.05 was considered as not statistically significant.

In the pro-inflammatory group, the release of M-CSF was significantly (*p* < 0.05) increased on day 2 and day 4. However, IL-1α levels were only significantly higher at day 4. The early release of IL-1α has been shown to play a major regulatory function in the expression of the master cytokine IL-1β after exposure to silica microparticles as well as nanoparticles in mice.^95^ However, it is important to note that no significant change was found in IL-1β release at both time points following exposure to SiC NWs, which could be because IL-1β release occurs as a late response requiring more than 4 days of exposure. This can be explained since silica-mediated IL-1β release is regulated through a canonical process via activation of the NLRP3 inflammasome, followed by conversion of pro-IL-1β into mature IL-1β and eventually Gasdermin D pore formation in the cell membrane that allows passage of IL-1β to the extracellular space.^96,97^ Furthermore, SiC NWs suppressed the secretion of IL-6 at both time points (days 2 and 4), potentially suggesting that a decrease in anti-inflammatory function of cells will further elicit the propagation of inflammatory signals in the cells.^98^ Furthermore, a transient increase in IL-17E (also called IL25) and a decrease in IL3 and IFN-α2 were detected on day 2, which normalized on day 4.

The IL-6-mediated anti-inflammatory signaling (known as *classical signaling*) is mainly driven by the membrane-bound IL-6 receptor (mIL-6R), where free extracellular IL-6 binds to mIL-6R and activates glycoprotein 130 (gp130, ubiquitously expressed in all cell types), triggering a downstream anti-inflammatory cascade.^99^ However, it is important to note that not all cell types express mIL-6R, and the most common cell types with mIL-6R expression are immune cells and hepatocytes.^100^ On the other hand, IL-6-mediated pro-inflammatory signaling is driven by the soluble IL-6 receptor (sIL-6R, known as *trans-signaling*), where IL-6 forms a complex with sIL-6R extracellularly, followed by the activation of gp130 and induction of a pro-inflammatory cascade in the cells. The observed decrease in the extracellular IL-6 after SiC NWs exposure indicates a potential complexation of IL-6 with sIL-6R (therefore not present in free form) that may eventually activate pro-inflammatory *trans-signaling*.

For the pro-fibrotic markers (Figure 6c), a significant (*p* < 0.05) induction in the release of platelet-derived growth factor (PDGF)-AA, chemokine ligand (CXCL)-9, FMS-like tyrosine kinase-3 Ligand (FLT-3L) and fibroblast growth factor 2 (FGF-2) was detected on both day 2 and day 4. PDGF-AA, FGF-2, and FTL-3L are cytokines involved in the proliferation and differentiation of cells. PDGF-AA has been demonstrated to be involved in mediating key functions in the pathogenesis of fibrosis, such as enhanced myofibroblast proliferation and chemotaxis, promoting collagen production, and mediating cell adhesion.^101,102^ CXCL-9 has been shown to abrogate TGF-β induced pulmonary fibrosis development by inhibiting epithelial-to-mesenchymal cell transition,^103^ Similarly, FGF-2 has been proposed as an upstream regulator in pulmonary fibrosis, since it can inhibit fibrotic gene expression related to the differentiation of fibroblasts into myofibroblasts.^104,105^ Taken together, the increased secretion of CXCL-9 and FGF-2 after exposure to SiC NWs could play a protective function against fibrosis development in pHBE cell cultures. An increased release of FLT3L has also been shown during lung fibrosis. The role of FLT3L is mainly associated with dendritic cell (DC) accumulation and mobilization into CD11b+ DCs, which limits the disease severity and development.^106^ Furthermore, IL-9 and G-CSF secretion were significantly (*p* < 0.05) suppressed on day 2, whereas no significant changes were found on day 4. The increased IL-9 and G-CSF secretion (or supplementation from outside) have been shown earlier to inhibit pulmonary fibrosis, suggesting their antifibrotic role in the lungs.^107–110^ Therefore, IL-9 and G-CSF suppression after SiC NWs exposure at the early time point (day 2) may indicate a pro-fibrotic potential of SiC NWs in lung epithelial cells.

Reference materials (SiO_2_ NPs, quartz DQ12, graphene nanosheets, and MWCNTs) also modulated cytokine-chemokine production to a different extent than the SiC NWs (SI-Table S3). In the pro-inflammatory and anti-inflammatory cytokine group, SiO_2_ NPs significantly reduced the release of IL-3 at day 2, while they increased the release of M-CSF. The elevated release of M-CSF was persistent until day 4, and was accompanied by elevated levels of exotain, FLT-3L, IFNγ, and GROα. Notably, the secretion of the anti-inflammatory cytokine IL-1RA was also increased on both day 2 and day 4 after exposure to SiO_2_ NPs. Surprisingly, quartz DQ12 only triggered a significantly (*p* < 0.05) higher release of GROα at day 2. In addition, a moderate induction in release of eotaxin, IL-8, and IL-10 at day 2 or MCP3, eotaxin, IL-17A, IL-3, and sCD-40L at day 4 was recorded (SI-Figure S10). However, these results were not statistically significant (*p* > 0.05), likely due to high variability across three independent measurements or an insufficient delivered cellular dose of quartz DQ12, which may have been removed by mucociliary clearance, failing to elicit a strong pro-inflammatory or pro-fibrotic response. Previous studies have also shown that crystalline silica-mediated inflammatory and fibrotic responses typically require prolonged (subchronic) and relatively high dose exposure, which could explain the moderate effects of quartz DQ12 observed in the present study.^67,111^ Furthermore, exposure to graphene nanosheets decreased the release of IL-2, MDC (CCL22), and IL-9 on day 2, but increased the secretion of G-CSF, IL-1α, and MIP-1β on day 4. Notably, IL-9 also functions as a pro-fibrotic cytokine.^112^ The decrease in the release of MDC was maintained on day 4 as well. MWCNTs elevated the release of FLT-3L, MDC, RANTES, and TNFα on day 2, but no significant effects were observed on day 4.

Previous studies have reported that NMs could trigger immunomodulatory and fibrotic responses in lung cells. Mukherjee et al. have shown that short-term (48 h) and high-dose (80 μg/mL) exposure to graphene oxide (GO) in human lung cells (BEAS-2B) affected fibrotic pathways and transcripts related to pro-fibrotic factors.^41^ In addition, authors could demonstrate enhanced collagen secretion and deposition after exposure to high doses of GO. Chortarea et al. investigated pro-inflammatory responses of MWCNTs in relation to quartz DQ12 after repeated exposure (5 days per week at 10 μg/cm^2^) for 5 weeks in pHBE cell cultures (from healthy and asthmatic donors). Their results, based on gene expression analysis, showed an increase in transcripts related to pro-inflammatory response (e.g., IL-8, IL-6, IP-10) following quartz DQ12 exposure, but no such effects were evident in the case of MWCNTs. In contrast, another study led by the same authors suggested that prolonged (96 h) exposure to MWCNTs (Mitsui-7, up to 20 μg/mL) resulted in a pro-inflammatory response by an increase in the secretion of TGF-β, PDGF, and osteopontin. The major difference in this study was that a cell line-based cell culture model (THP1, A549, and MRC-5) was used, which may not as closely reflect lung epithelial morphological complexity as the pHBE cell cultures. Taken together, these findings highlight the potential importance of the cell culture model complexity in nanosafety assessment.

Li et al. showed that SiO_2_ NPs exposure in THP1 macrophages elicited the release of pro-inflammatory cytokines (IL-β, TGF-β, and TNF-α). Next, the authors could demonstrate that exposure of lung fibroblast cells (MRC-5 cells) to the conditioned medium from SiO_2_ NPs induced TPH1 macrophages to stimulate transdifferentiation of fibroblasts into myofibroblast-like cells. In a recent *in vivo* study, Li et al. performed single-cell RNAseq on rat lungs after exposure to silica NPs.^113^ Their results showed the induction of proteostasis and immunomodulatory effects in silica NP-exposed rat lung cells that elicited fibroblast proliferation and secretion of extracellular matrix proteins and contributed to the development of pulmonary fibrosis.

Mechanistically (as illustrated in Figure 7), the observed biological effects in this study suggest that SiC NWs passed through the mucus barrier into the extracellular ciliary region and caused ciliary damage and mislocalization of basal bodies. Basal bodies play a crucial role in anchoring respiratory cilia into plasma membranes and maintaining their motility to perform mucous clearance functions.^114^ Therefore, the observed defects in basal bodies could potentially be the main cause of ciliary dysfunction. Moreover, the subpopulation of SiC NWs with a smaller size and shorter length was potentially taken up by the epithelial cells (SiC NWs of 0.5–5 μm length found within cells, Figure 5b) and modulated certain inflammatory and fibrotic cytokines/chemokines in pHBE cell cultures. Inflammatory responses in pHBE cells may further lead to increased mucus production. To counteract the SiC NWs-mediated effects and maintain normal ciliary functions (e.g., mucociliary clearance), expression of genes related to the ciliogenesis pathway was activated (upregulation of *FOXJ1* and *CCP110*) that could promote proliferation and differentiation of basal cells into ciliated cells. As a result, more newly generated, thin cilia were observed by TEM after SiC NWs exposure (Figure 5c).

**Figure 7:**
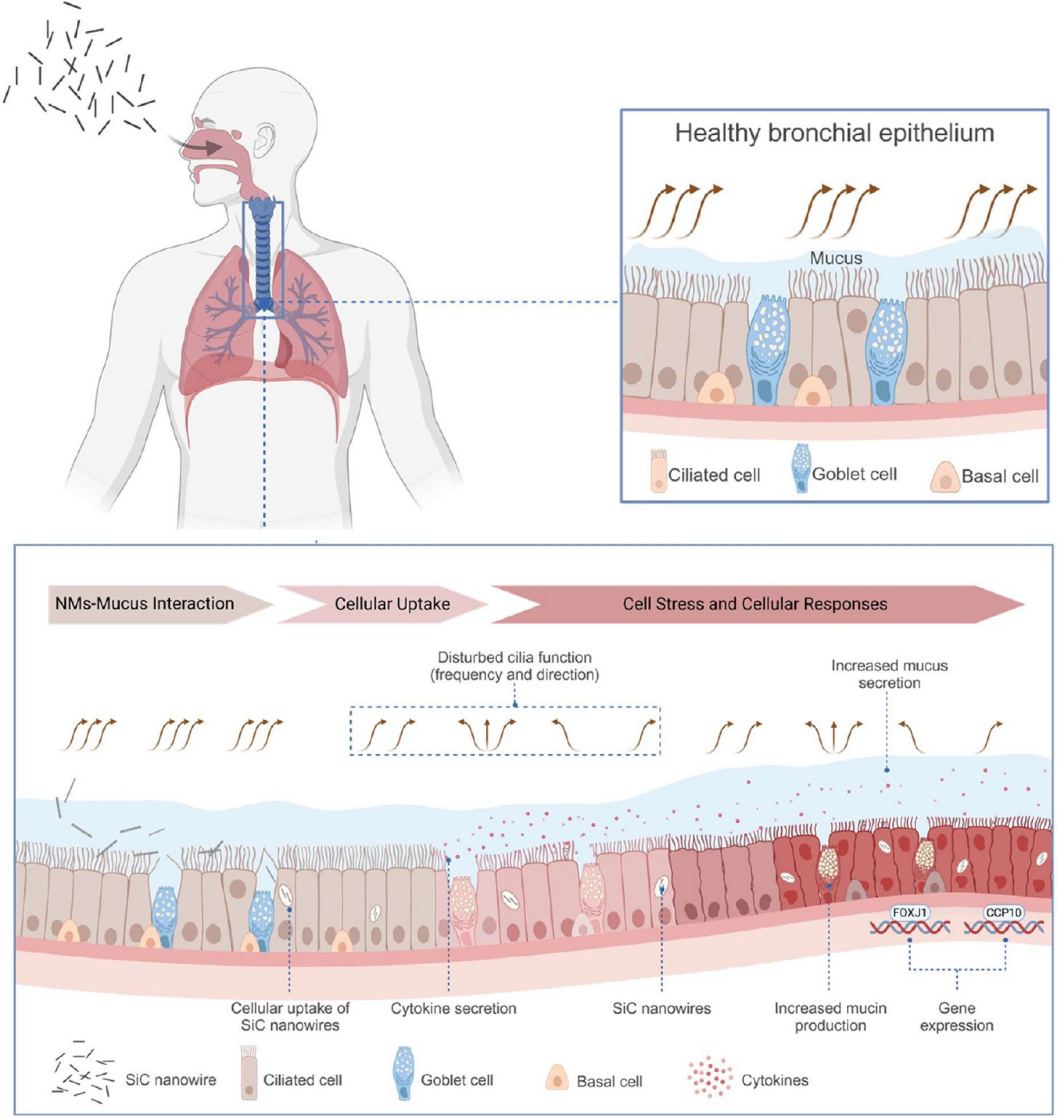
Mechanistic illustration showing the potential effects of SiC NWs on the pHBE cell cultures after 4 days of repetitive exposure under ALI conditions. The model shown in the top right box depicts a healthy state of respiratory epithelium with unidirectional movement of motile cilia performing mucociliary clearance function. Illustrated model at the bottom shows a sequential progression of biological responses over time after exposure to SiC NWs. The inhalation exposure of SiC NWs in pHBE cell cultures did not interact with respiratory mucus, thus penetrated through the mucus layer and reached the periciliary region. Once in the periciliary regions, it causes ciliary damage and basal body mislocalization from the plasma membrane of ciliated epithelial cells, resulting in reduced mucociliary clearance. Next, SiC NWs with smaller size and length are taken up by the cells and trigger inflammatory and profibrotic cytokine-chemokine release that potentially leads to increased mucus secretion from the goblet cells. As a protective function to the SiC NWs exposure, the increase in the mRNA expression of genes related to ciliogenesis and mucus production is observed in pHBE cell cultures. The figure was created with BioRender.com.

## Conclusions

Our study investigated the extracellular and intracellular interaction of SiC NWs, SiO_2_ NPs, quartz DQ12, graphene, and MWCNTs with human bronchial mucus and primary human bronchial epithelial cell cultures. Results showed that SiC NWs, unlike the other materials, were not mucoadhesive and could penetrate through the airway mucus barrier. This results in subtoxic effects on crucial ciliary functions and triggers the release of inflammatory and fibrotic cytokines. In contrast, other NMs reached the cells with delayed arrival or lower quantities, which were insufficient to trigger significant adverse responses, though minor effects on TEER value and specific cytokines and chemokines were evident. These findings provide the first evidence that SiC NWs can impair upper respiratory tract innate immune defense by impacting mucociliary clearance function. This underscores the necessity of further investigating the inhalation toxicity of these advanced materials, particularly in the alveolar region. Future research should focus on the long-term effects of SiC NWs exposure and potentially explore “Safe and Sustainable by Design (SSbD)″ strategies to mitigate their potential human hazard. SiC NWs exposure in combination with secondary pollutants and/or pathogens should also be explored in the future since it can piggyback them in lung cells, and that may pose more severe damages compared to individual exposures.

## Materials and Methods

### Materials Preparation and Dispersion

Silicon carbide nanowires (SiC NWs; purchased from ACS Material, D: 100–1000 nm, L: 10–50 μm, Purity: 80–90%, CAS No. 1568-80-5), multiwalled carbon nanotubes (MWCNTs, obtained from JRC, product name NM401), SiO_2_ NPs (obtained from JRC, product name NM203), Graphene (obtained from JRC, product name JRCNM48001a) and silica quartz (DQ12, provided by Dr. Martin Weimann from IBE) were used in the study. All of the materials were obtained initially in powder form and then dispersed either in endotoxin-free ultrapure water (CAS No. TMS-011-A, Sigma-Aldrich) for characterization experiments or in 0.05% BSA-water for cell culture experiments using probe sonication. SiO_2_ NPs, quartz DQ12, and MWCNTs were dispersed using a probe sonicator (6% amplitude) for 16 min in ice-cold water by following the so-called Nanogentox protocol (https://www.anses.fr/en/system/files/nanogenotox_deliverable_5.pdf). However, SiC NWs were dispersed using a probe sonicator (10% amplitude) for 1 min. Graphene nanosheets were dispersed in 0.05% BSA-water by mild vortexing for 2 min, followed by a 45 min water bath sonication in two intermittent cycles (Cycle 1 = 20 min and Cycle 2 = 25 min). Stock solutions were then diluted to achieve working concentrations (1, 10, 25, and 50 μg/cm^2^) in 0.9% sterile NaCl saline. Cell culture exposed to 0.05% BSA-water in 0.9% NaCl was used as a vehicle control.

### Characterization of Materials before and after Interaction with Airway Mucus

100 μL (1 mg) from each of the dispersed materials (10 mg/mL)­was incubated with 50 μL of undiluted airway bronchial mucus (collected after culturing pHBE cell cultures) at 37 °C for 2 h.^115^ Following incubation, 850 μL of Milli-Q water was added, and the mixture was centrifuged at 20,000*g* at 4 °C for 30 min. The pellet was kept and redispersed in 500 μL of Milli-Q water by thorough vortexing and used for physicochemical characterization as below. Pristine materials and mucus alone were also characterized in parallel to compare potential changes in material properties after incubation with airway mucus.

#### Hydrodynamic Size and ζ Potential

The NM hydrodynamic size and ζ potential were determined by using a Zetasizer Ultra instrument (Malvern Instruments, UK). NM dispersion was vortexed for 1 min to minimize agglomeration. Size measurements were conducted at 37 ± 0.1 °C. For the ζ potential, the instrument calibration was performed with a standard polystyrene latex prior to measurements, and the measurements were conducted at 25 ± 0.1 °C. Fifteen repeated measurements were performed for each sample.

#### Raman Confocal Scanning

Raman confocal spectroscopy was employed to analyze the interaction between NMs and the mucus components. SiC NWs, graphene, and MWCNTs were prepared on glass slides, while SiO_2_ NPs and quartz DQ12 were prepared on copper tape attached to glass slides. Raman spectra were acquired at 100× objective lens in room temperature (22 ± 1 °C) using a confocal Raman microscope equipped with 532 and 488 nm lasers (WITec alpha300 RAS system, Oxford Instruments, Germany). The acquisition parameters for each material are provided in SI-Table S4. All spectra were analyzed by using WITec Project or LabSpec software. Baseline correction and normalization were performed to account for any background noise.

#### Fourier Transform Infrared

Fourier-transform infrared (FTIR) spectroscopy was conducted to identify functional groups on the material surfaces. Spectra were recorded at ATR mode using a Bruker Tensor 27 IR spectrometer in the range of 4000–600 cm^–1^ with a resolution of 4 cm^–1^, averaging 32 scans per sample. Background correction was performed with Milli-Q water prior to each measurement to minimize interference. Results were processed using OPUS 8.5 software (Bruker, Germany).

#### Contact Angle Measurement

Contact angle measurements were performed to evaluate the hydrophilicity and wettability of the material surfaces. NMs were dispersed in Milli-Q water. Ten μL of NM dispersion was dropped on a glass slide and air-dried in a chemical hood. Measurements were conducted by using a DSA25 Drop Shape Analyzer (KRÜSS Scientific GmbH, Hamburg, Germany) and DSA25 software for angle analysis. A 2 μL droplet of deionized water was carefully dispensed onto the surface using a microsyringe to minimize impact forces. Images of the droplet were captured immediately after deposition to avoid evaporation effects. The contact angle was determined using the sessile drop method, with angles measured on both sides of the droplet to ensure consistency. Six replicates were performed on each NM. For accuracy, the baseline was adjusted manually where necessary. All measurements were conducted at room temperature (22 ± 1 °C). Data were presented as mean ± SD. The results were used to infer surface hydrophilicity, with lower contact angles indicating higher wettability.

#### Transmission Electron Microscopy

The samples for TEM analysis were generally prepared by drop-casting 5 μL of material suspensions with or without mucus components for 1 min onto a 200-mesh copper grid with holey or continuous carbon film (Electron Microscopy Resolutions, HC200Cu100 or C200Cu100). For SiO_2_ NPs, quartz DQ12 and MWCNTs, the carbon grids were pretreated with a 3 min-incubation on a 300 μL droplet of 0.1% (w/v) poly-l-lysine solution (PLL, P8920, Sigma-Aldrich) and subsequent blotting on Whatman filter paper for grid hydrophilization and enhanced sample adsorption. Moreover, 5 μL of SiO_2_ NPs was incubated for 2 min, while 10 μL of the quartz DQ12 and MWCNTs samples were incubated for 2 min on the carbon grids for better sample dispersion. After the excess sample was blotted away for all grids with Whatman filter paper, the grids of materials without mucus incubation were air-dried overnight at room temperature. The grids with materials and mucus were instead negatively stained by incubating 5 μL of 2% phosphotungstic acid (PTA, pH 7.5) for 1 min, then blotted as above, and air-dried. The grids with and without mucus were imaged using a Zeiss EM 900 microscope at 80 kV (Carl Zeiss Microscopy GmbH, Germany) and different magnifications.

### Cell Culture and Exposure

MucilAir pHBE cell cultures (EP01MD, Epithelix) were maintained in MucilAir culture medium (EP04MM, Epithelix) with 5% CO_2_, 100% humidity at 37 °C. Materials dispersion of 10 μL at the concentration of 1, 10, 25, and 50 μg/cm^2^ was added to the apical compartments once a day for four consecutive days. After 4 days, the samples (cell cultures and basolateral supernatant) were collected for different assays.

### Ciliary Interaction and Cellular Uptake of Materials in pHBE Cell Cultures

#### Scanning Electron Microscopy

The interaction of materials with the cell surface and ciliary morphology of pHBE cell cultures was determined using SEM. Following 4 days of repetitive exposure (10 μg/cm^2^) to SiC NWs, SiO_2_ NPs, MWCNTs, Graphene, and quartz DQ12, pHBE cell cultures were washed twice with sterile 1× PBS (Gibco) in the transwell inserts. Subsequently, the pHBE cell cultures were fixed with 3% glutaraldehyde (Sigma-Aldrich, Germany) prepared in 0.1 M Na-cacodylate buffer (Electron Microscopy Sciences, pH 7.4) for 25 min at room temperature (RT), followed by repetition with fresh fixative for 35 min at 4 °C. The samples were then washed twice for 20 min at 4 °C with 0.2 M Na-cacodylate buffer (Electron Microscopy Sciences, pH 7.4) and kept in fresh 0.2 M Na-cacodylate buffer at 4 °C until further processing for SEM or TEM. For SEM, the samples were next subjected to an Ethanol (HoneyWell, Riedel-de-Haen) dehydration series (30 min 50%, 30 min 70%, 30 min 80%, 60 min 90% and 60 min 100% Ethanol at RT) and incubated with hexamethyldisilazane (HMDSO, 205389, Sigma-Aldrich, Germany) for 30 min at RT. After air-drying the transwells overnight at RT, the membranes were cut out from the transwell inserts using a scalpel, glued onto SEM stubs, and sputter-coated with 10 nm carbon (high vacuum coater Leica EM ACE 600, Switzerland). Images of the samples’ cilia surface were acquired using an Axia ChemiSEM (Thermo Fisher Scientific) microscope at 10 kV, 16,000× magnification, and 65 pA with an ETD detector.

#### Transmission Electron Microscopy

The presence of materials in cells and materials-induced ultrastructural changes in pHBE cell cultures were determined using TEM. To this end, the cells on transwell inserts were cultured, fixed, and stored in a cacodylate buffer as for SEM described above. Then, the membranes with adhering cells were excised from the transwell inserts for TEM using a scalpel, and stained with 2% OsO4 (Electron Microscopy Sciences) in 0.1 M Na-cacodylate buffer for 2 h at 4 °C in a glass vial, as adapted from Gupta et al. The membranes were then washed twice with Milli-Q water, once for 7 min at RT and once for 3 min at 4 °C. Afterward, the membranes were serially dehydrated using an ethanol gradient (10 min 50%, 10 min 75%, 2 × 15 min 100% EtOH from HoneyWell, Riedel-de-Haen, then 3× 30 min 100% water-free EtOH from Sigma-Aldrich) at 4 °C. Next, the membranes holding pHBE cell cultures were shortly incubated in 100% acetone (Sigma-Aldrich, Germany) at RT, followed by an Epon gradient in acetone (33% Epon at 4 °C overnight, 66% at 4 °C for 6 h, 100% at RT for 2 h with the glass vial lid open for acetone evaporation) using Epon 812 substitute resin (Epoxy embedding kit 45359, Sigma-Aldrich, Germany). Finally, the transwell membranes holding pHBE cell cultures were cut into smaller pieces using scissors, embedded in molds using fresh 100% Epon as above, and cured at 60 °C for at least 2 days. For TEM imaging, ultrathin sections ranging from 80 to 100 nm in thickness were prepared using an ultramicrotome (Leica EM UC6, Germany), placed onto Formvar-coated copper grids (100 mesh, EM Resolutions), air-dried, and then imaged at different magnifications with a Zeiss EM 900 microscope (Carl Zeiss Microscopy GmbH, Germany) at 80 kV. Notably, the samples were only fixed with glutaraldehyde and stained with osmium tetroxide, and the sections were not additionally poststained with uranyl acetate or lead citrate to minimize the potential formation of stain precipitates that could be mistaken for the NMs studied here. Nonetheless, osmium staining provided a sufficient contrast for cellular ultrastructure visualization. Internalized SiC NWs were measured in their length by using ImageJ.

### Cytotoxicity Assays

#### Lactate Dehydrogenase (LDH) Release Assay

LDH is a cytoplasmic enzyme that is commonly used as a marker of cell membrane integrity. The release of LDH into the cell culture medium is indicative of the plasma membrane rupture. Triton-X-100 (10%) was applied apically for 24 h as a positive control. Subsequently, 50 μL of cell culture medium from the basolateral compartment was collected on days 2 and 4 for the LDH release assay, respectively. LDH release assay was performed with a Cytotoxicity LDH Assay Kit-WST (Dojindo, CK 12–20, TU797, Japan). The absorbance at 490 nm was recorded with a microplate reader (Victor Nivo Microplate Reader, PerkinElmer Inc., USA). Absorbance was corrected with a blank control (cell culture medium only). Results are shown as a percentage of viable cells compared to the highest LDH release in the positive control. The experiments were carried out with three biological replicates.

#### Trans-Epithelial Electrical Resistance (TEER) Measurement

TEER is a measurement method for evaluating the epithelial barrier function. This dynamic parameter reflects the state of epithelia and is typically between 200 and 800 Ω·cm^2^ for MucilAir. When an epithelium is damaged, a decrease in TEER would be associated with an increase in LDH release or a decrease in cell viability. The threshold TEER value for epithelial disruption is 100 Ω·cm^2^. The resistance was measured using an STX2 electrode with an EVOMX volt-ohmmeter (World Precision Instruments UK, Stevenage) after the addition of 200 μL saline solution to the apical compartment of the cultures (which was removed quickly afterward). Resistance values (Ω) were converted to TEER values (Ω.cm^2^) using the following formula: TEER (Ω·cm^2^) = (resistance value (Ω) – 100­(Ω)) × 0.33 (cm^2^), where 100 Ω is the resistance of the membrane and 0.33 cm^2^ is the total surface of the epithelium.

#### Cilia Beating Frequency (CBF)

CBF measured in hertz (Hz) was determined using a Sony XCD V60 camera coupled to an Olympus BX51 microscope and a PCI card. A total of 256 images were captured at a frequency of 125 frames per second at room temperature. Subsequently, CBF was analyzed and calculated using CiliaX software (Epithelix).

#### Mucociliary Clearance (MCC)

MCC was monitored with a Sony XCD-U100CR camera connected to an Olympus BX51 microscope with a 5× objective lens. Polystyrene microbeads of 30 μm diameter (Sigma, 84135) were applied to the apical surface of MucilAir pHBE cultures. The movement of microbeads was recorded at a rate of 4 frames per second, capturing 60 images at room temperature. Three separate videos were recorded for each insert. The average velocity of bead movement (μm/s) was calculated using the ImageProPlus 6.0 software.

### Cytokine-Chemokine Assays

#### IL-8 ELISA

Samples were diluted 1:1000 with the appropriate assay diluent, and Interleukin 8 (CXCL8, IL-8) release was quantified using an ELISA kit (BD Biosciences 555244) following the manufacturer’s protocol. Each ELISA plate included a standard curve, and washing steps were carried out using an automatic microplate washer (405 TS, Biotek Instruments, USA). Absorbance was recorded at 450 nm with a plate reader (Victor Nivo Microplate Reader, PerkinElmer Inc., USA).

#### Multiplexed Cytokine Array

The release of cytokine/chemokine pHBE cell cultures in the basolateral medium after exposure to SiC NWs, SiO_2_ NPs, MWCNTs, graphene, and quartz DQ12 for 2 and 4 days was analyzed by Luminex based Human Cytokine/Chemokine Panel A 48-Plex Discovery Assay (HD48A, Eve Technologies Corp, Calgary, Canada). The 48-plex array consisted of the following cytokines and chemokines: sCD40L, EGF, Eotaxin, FGF-2, Flt-3 ligand, Fractalkine, G-CSF, GM-CSF, GROα, IFNα2, IFNγ, IL-1α, IL-1β, IL-1ra, IL-2, IL-3, IL-4, IL-5, IL-6, IL-7, IL-8, IL-9, IL-10, IL-12p40, IL-12p70, IL-13, IL-15, IL-17A, IL-17E/IL-25, IL-17F, IL-18, IL-22, IL-27, IP-10, MCP-1, MCP-3, M-CSF, MDC (CCL22), MIG, MIP-1α, MIP-1β, PDGF-AA, PDGF-AB/BB, RANTES, TGFα, TNFα, TNFβ, VEGF-A. For the assay, the basolateral medium was collected and centrifuged (500*g*, 4 °C, 20 min), followed by passing through a microcolumn filter (pore size: 0.22 μm) to remove any residual cellular debris. The conditioned medium from cells exposed to CytoMix (consisting of 0.5 μg/mL of TNF-α (Biolegend, catalogue no. 570104), 200 μg/mL of LPS (Sigma, catalogue no. L9143), and 1% fetal calf serum (Bioconcept, catalogue no. 2-01F16–I)) was used as the positive control. The supernatant was stored at −80 °C until further use. The analysis of each sample was done in duplicate, and no further dilutions were performed. For data analysis and curation, the mean values from two replicates per sample were considered. *Z*-scored row entries were visualized as a heat map using the pheatmap library (version 1.0.12, Kolde, R. pheatmap: Pretty Heatmaps, 2022).

#### mRNA Expression Analysis: RNA Isolation, cDNA Synthesis, and qPCR

Following 4 days of repeated exposure, the transwell membrane of each pHBE cell culture was excised and placed into separate 1 mL Eppendorf tubes. Subsequently, 200 μL of RLT lysis buffer (QIAGEN GmbH, 79216) was added to each tube. Nuclease-free cell pestles were used to facilitate the rupturing of the tissue. RNA was isolated using the RNeasy Micro Kit (QIAGEN GmbH, Germany, 74004) following the manufacturer’s protocol. The purity and yield of RNA isolates were analyzed before further processing using a NanoDrop One (ThermoFisherScientific)

cDNA synthesis was performed with an iScript cDNA Synthesis Kit (Bio-Rad, USA, 1708891) by following instructions from the manufacturer. qPCR analysis was performed on PrimePCR Custom 96-well plates (Bio-Rad, USA; gene name and unique ID are provided in SI-Table S2) using SsoAdvanced Universal SYBR Green Supermix (Bio-Rad, 1725271) following the manufacturer’s protocol. Results were analyzed and plotted with GraphPad Prism 10 (GraphPad Software, USA). For comparison of expression levels, comparative CT analysis (ΔΔCT method) was employed with GAPDH as a housekeeping gene. Expressions were normalized against the control.

### Statistical Analysis

Statistical analysis was performed and presented with GraphPad Prism 10 (GraphPad Software, USA). Details of the statistical methods used for each specific data set are described in the corresponding figure legends.

## Supplementary Material









## Data Availability

All data necessary to support the conclusions of this study are included within the paper and its Supporting Information. Additional data sets related to this research are available from the corresponding authors upon reasonable request.

## Notes

Tables summarizing the physicochemical properties of materials; parameters applied in Raman analysis of materials; list of genes and their unique assay IDs for qPCR; SEM and TEM images of pHBE cell cultures; characterization results of airway mucus, SEM images showing ciliary morphology, TEM images showing cellular uptake, ciliary function results; epithelial barrier integrity and cell viability results; and IL-8 cytokine release and cytokine-chemokine array results (PDF)
  Unidirectional movement of polystyrene beads was clearly visible in control cell cultures (AVI)
  SiC NWs-exposed cultures either showed a slow and nonunidirectional movement of beads at low dose exposure (10 μg/cm^2^) (AVI)
  A nearly complete loss in movement at a higher exposure dose (50 μg/cm^2^) (AVI)
