# Supplementary material for: Silicon Carbide Nanowires Impair Mucociliary Clearance-Mediated Innate Immunity in Primary Human Bronchial Epithelial Cells
Source: ACS Nano. 2025 Jun 6;19(23):21426–45. doi: 10.1021/acsnano.5c01981 (PMC12177949; doi:10.1021/acsnano.5c01981)
Supplement: Supplementary file 1 [file nn5c01981_si_001.pdf]

Supplementary Information for

Silicon Carbide Nanowires Impair Mucociliary Clearance-Mediated Innate Immunity in Primary Human Bronchial Epithelial Cells

Ziting Wang[1], Jimmy Vernaz[2], Nikolaos Tagaras[1], Bernadett Boda[2], Tina Buerki-Thurnherr[1], Giacomo Reina[1], Vera M. Kissling[1], Samuel Constant[2], Govind Gupta[1]*, Peter Wick[1] *

[1]Nanomaterials in Health Laboratory, Department of Materials Meet Life, Swiss Federal Laboratories for Materials Science and Technology (Empa), CH-9014 St. Gallen, Switzerland

[2]Epithelix Sàrl, 18 chemin des Aulx, 1228, Plan-les-Ouates, Switzerland

*Corresponding authors

Peter Wick
E-mail: peter.wick@empa.ch
Orcid ID: 000-0002-0079-4344

Govind Gupta
E-mail: govind.gupta@empa.ch
Orcid ID: 0000-0003-4703-418X

Table S1: Summary of the physicochemical properties of the materials.

Product Name	Chemical name	CAS Number	Specific surface area (m^2/g)	Average Length (μm)	Average Diameter (nm)	Surface Chemistry
SiC NWs[1]	Silicon carbide	1568-80-5	*NA	10-50	100-1000	NA
DQ12[2]	NA	14808-60-7	1.5	#NR	560 (number weighted)	NA
NM203-JRCNM10404[3]	Silicon dioxide NPs	112945-52-5	198 (40°C) 195 (-80°C)	NR	48±4	71.7% O; 26.0% Si; 2.31% C (surface contamination)
NM401-JRCNM04001[4]	Multiwalled Carbon Nanotubes (MWCNTs)	7782-42-5	140	4.048	67	NA
Graphene-JRCNM48001a	Graphene nanosheets	7782-42-5	NA	NA	3892	NA

*NA - information not available. #NR – Information not relevant

Note: Information presented here for different particles is extracted from publicly available documents cited for respective particles.

[1]ACS Material Silicon Carbide Nanowires Technical Data Sheet. https://www.acsmaterial.com/pub/media/catalog/product/file/TDS-_NEW_Silicon_Carbide_Nanowire_102819.pdf?srsltid=AfmBOoqhgwC5uxheEAD48ntG3Tf3J6ru-HLcuZAHlsti9zkPfUreqd8L

[2]Ziemann, C.; Rittinghausen, S.; Ernst, H.; Kolling, A.; Mangelsdorf, I.; Creutzenberg, O. "Genotoxic Mode of Action of Fine and Ultrafine Dusts in Lungs"– Project F 2135 – on behalf of the Federal Institute for Occupational Safety and Health.

[3]Rasmussen K, Mech A, Mast J, De Temmerman P, Waegeneers N, Van Steen F, Pizzolon J, De Temmerman L, Van Doren E, Jensen K, Birkedal R, Levin M, Nielsen S, Koponen I, Clausen P, Kembouche Y, Thieriet N, Spalla O, Giuot C, Rousset D, Witschger O, Bau S, Bianchi B, Shivachev B, Gilliland D, Pianella F, Ceccone G, Cotogno G, Rauscher H, Gibson P, Stamm H. Synthetic Amorphous Silicon Dioxide (NM-200, NM-201, NM-202, NM-203, NM-204): Characterisation and Physico-Chemical Properties. EUR 26046. Luxembourg (Luxembourg):

Publications Office of the European Union; 2013. JRC83506. DOI: 10.2788/57989
[4]Rasmussen K, Mast J, De Temmerman P, Verleysen E, Waegeneers N, Van Steen F, Pizzolon J, De Temmerman L, Van Doren E, Jensen K, Birkedal R, Clausen P, Kembouche Y, Thieriet N, Spalla O, Giuot C, Rousset D, Witschger O, Bau S, Bianchi B, Shivachev B, Dimowa L, Nikolova R, Nihtianova D, Tarassov M, Petrov O, Bakardjieva S, Motzkus C, Labarraque G, Oster C, Cotogno G, Gaillard C. Multi-walled Carbon Nanotubes, NM-400, NM-401, NM-402, NM-403: Characterisation and Physico-Chemical Properties. EUR 26796. Luxembourg (Luxembourg): Publications Office of the European Union; 2014. JRC91205. DOI: 10.2788/10986

Table S2: Gene names and unique IDs for qPCR analysis.

Gene	Unique Assay ID
CCP110	qHsaCID0016944
CFTR	qHsaCID0018330
FOXJ1	qHsaCID0016777
KRT5	qHsaCED0005398
MKI67	qHsaCID0011882
MUC5AC	qHsaCID0017663
CCDC40	qHsaCED0045023
DNAH5	qHsaCID0017886
DNALI1	qHsaCID0007389
DYX1C1	qHsaCED0048914
RSPH4A	qHsaCID0017043
SPAG1	qHsaCED0043012
GAPDH	qHsaCED0038674

Table S3: Summary of cytokine-chemokine changes in response to exposure of reference materials in the pHBE cell cultures

Material	Pro-inflammatory biomarkers (day 2)	Pro-inflammatory biomarkers (day 4)	Pro-fibrotic biomarkers (day 2)	Pro-fibrotic biomarkers (day 4)	Other
SiO_2 NPs	IL-3 ↓<br>M-CSF ↑	Eotaxin ↑<br>FLT-3L ↑<br>GROα ↑<br>IFNγ ↑<br>M-CSF ↑	FGF-2 ↑<br>M-CSF ↑	Eotaxin ↑<br>M-CSF ↑	IL-1RA ↑<br>(day 2, day 4)<br>anti-inflammatory
DQ12	GROα ↑	ns	ns	ns	ns
Graphene	IL-2 ↓<br>IL-9 ↓	G-CSF ↑<br>IL-1α ↑	IL-9 ↓	FGF-2 ↑<br>IL-1α ↑	ns

	MDC (CCL22) ↓	MDC ↓<br>MIP-1β ↑			
MWCNTs	FLT-3L ↑<br>MDC ↑<br>RANTES ↑<br>TNF-α ↑	ns	TNF-α ↑<br>VEGF-A ↑	ns	ns

* ↑ – increased; ↓ – decreased; ns – no statistical significance.

Table S4: Experimental details on Confocal-Raman scanning

Sample	Scan width (μm)	Scan height (μm)	Points per Line	Lines per Image	Integration Time (s)	Excitation Wavelength (nm)	Laser Power (mW)
Mucus	50	50	50	50	0.5	532	15
Pristine SiC NWs	50	50	25	25	0.1	532	5
Mucus+SiC NWs	50	50	50	50	0.05	532	5
Pristine SiO_2 NPs	20	20	10	10	2	457	18
Mucus+SiO_2 NPs	20	20	10	10	2	457	18
Pristine DQ12	50	50	50	50	0.1	532	10
Mucus+DQ12	50	50	50	50	0.05	532	10
Pristine graphene	50	50	50	50	0.05	532	5
Mucus+graphene	50	50	50	50	0.05	532	5
Pristine MWCNTs	50	50	50	50	0.05	532	5
Mucus+MWCNTs	50	50	50	50	0.05	532	5

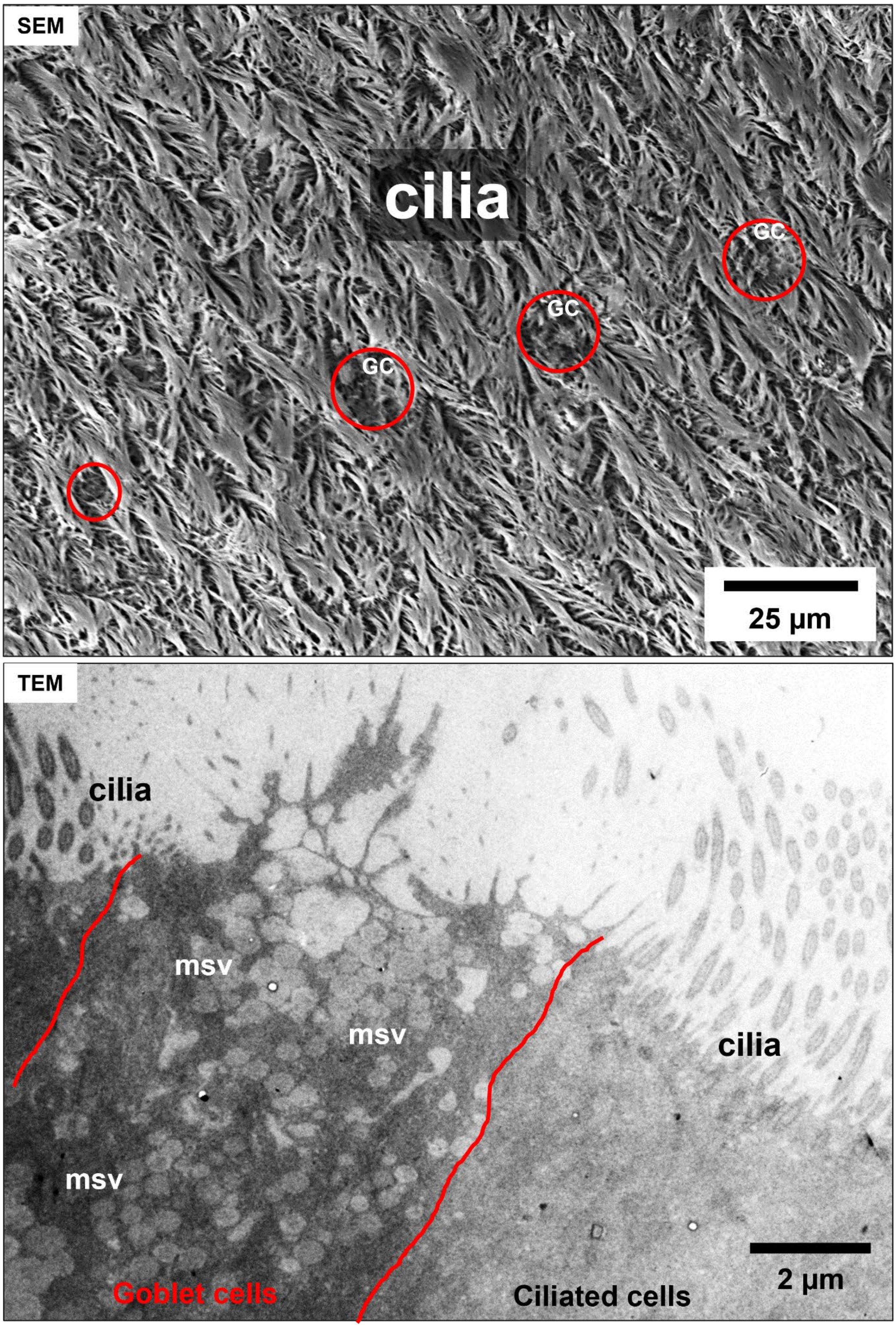

Figure S1: SEM and TEM images of pHBE cell cultures. Top: SEM image showing the surface view of a pHBE cell culture, with cilia (hairy structure) covering most of the epithelial surface and only few areas without cilia (red circles) indicative of goblet cells (GC). Bottom: TEM image showing the ultrastructure of a goblet cell filled with mucus-secreting vesicles (msv) and neighboring ciliated cells with cilia on the apical surface.

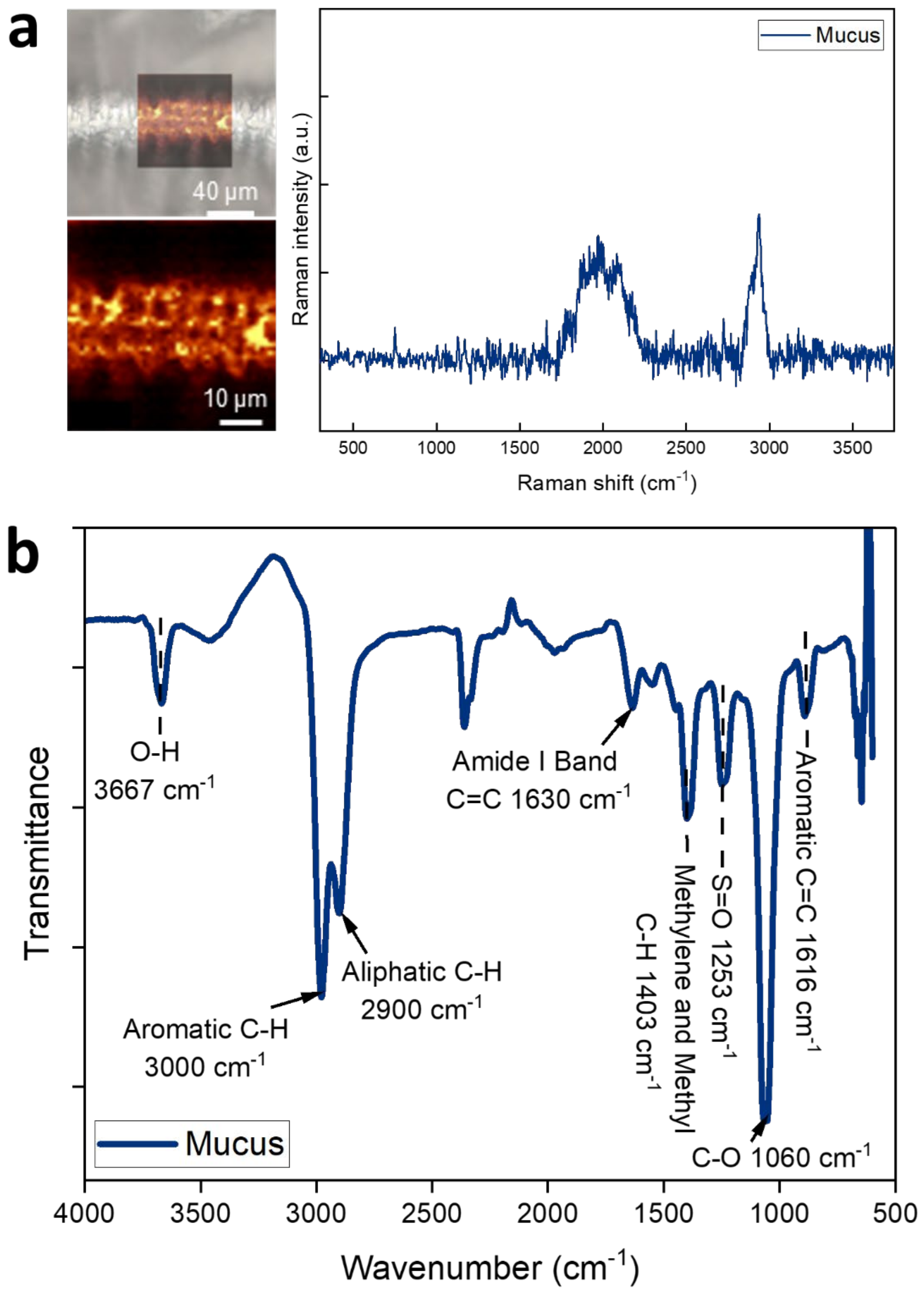

Figure S2: Characterization of airway mucus collected from HBE cell cultures. (a) Raman microscopy,and (b) FITR spectroscopy. a.u.: arbitrary units.

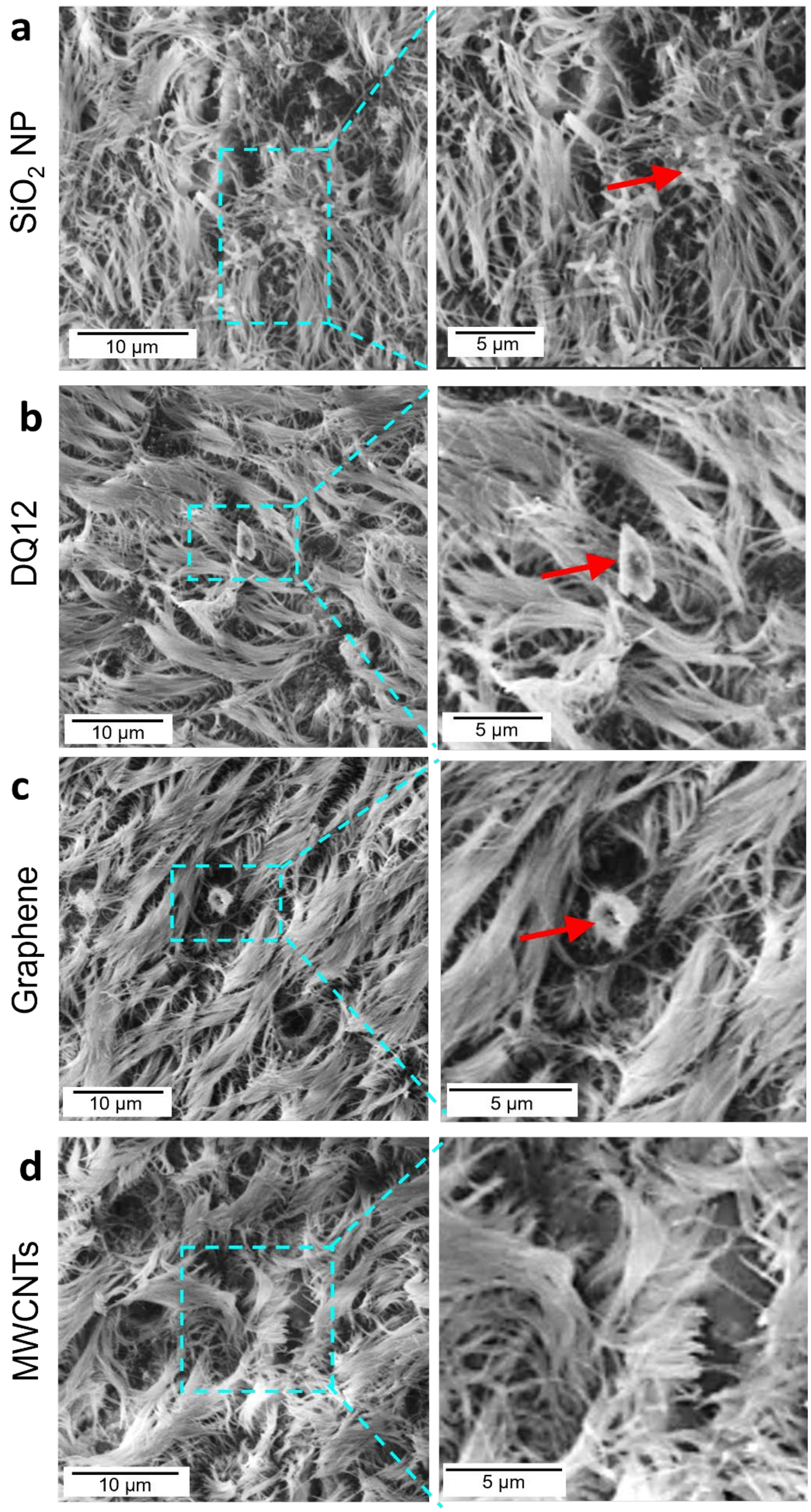

Figure S3: SEM images showing ciliary morphology of pHBE cell cultures after 4 days of exposure (10 µg/cm^2). (a) SiO_2 NPs, (b) DQ12, (c) graphene and (d) MWCNTs. Red arrows indicate respective materials in the periciliary region.

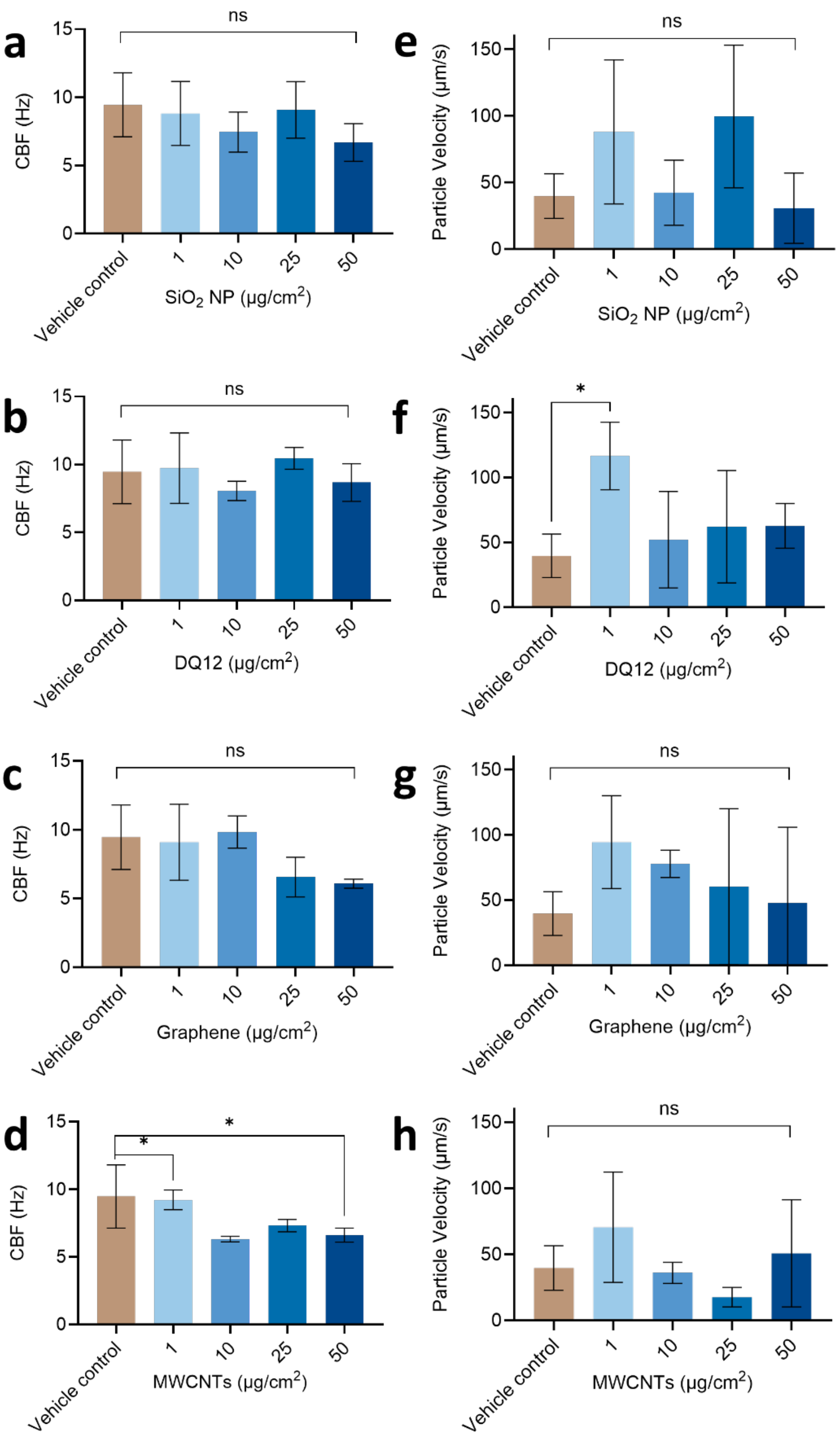

Figure S4: Ciliary function of pHBE cell cultures after 4 days of repetitive material exposure. CBF of pHBE cell cultures after exposure to (a) SiO_2 NPs, (b) DQ12, (c) graphene, and (d) MWCNTs. Mucociliary clearance function of the pHBE cell cultures after exposure to (e) SiO_2 NPs, (f) DQ12, (g) graphene, and (h) MWCNTs. Data presented as mean ± SD (n=3). *p*-value was calculated by applying ordinary One-way ANOVA and Dunnett's multiple comparison test for *post hoc* analysis. *$p < 0.05$ was considered statistically significant with respect to the control. $p > 0.05$ was considered as not statistically significant (ns).

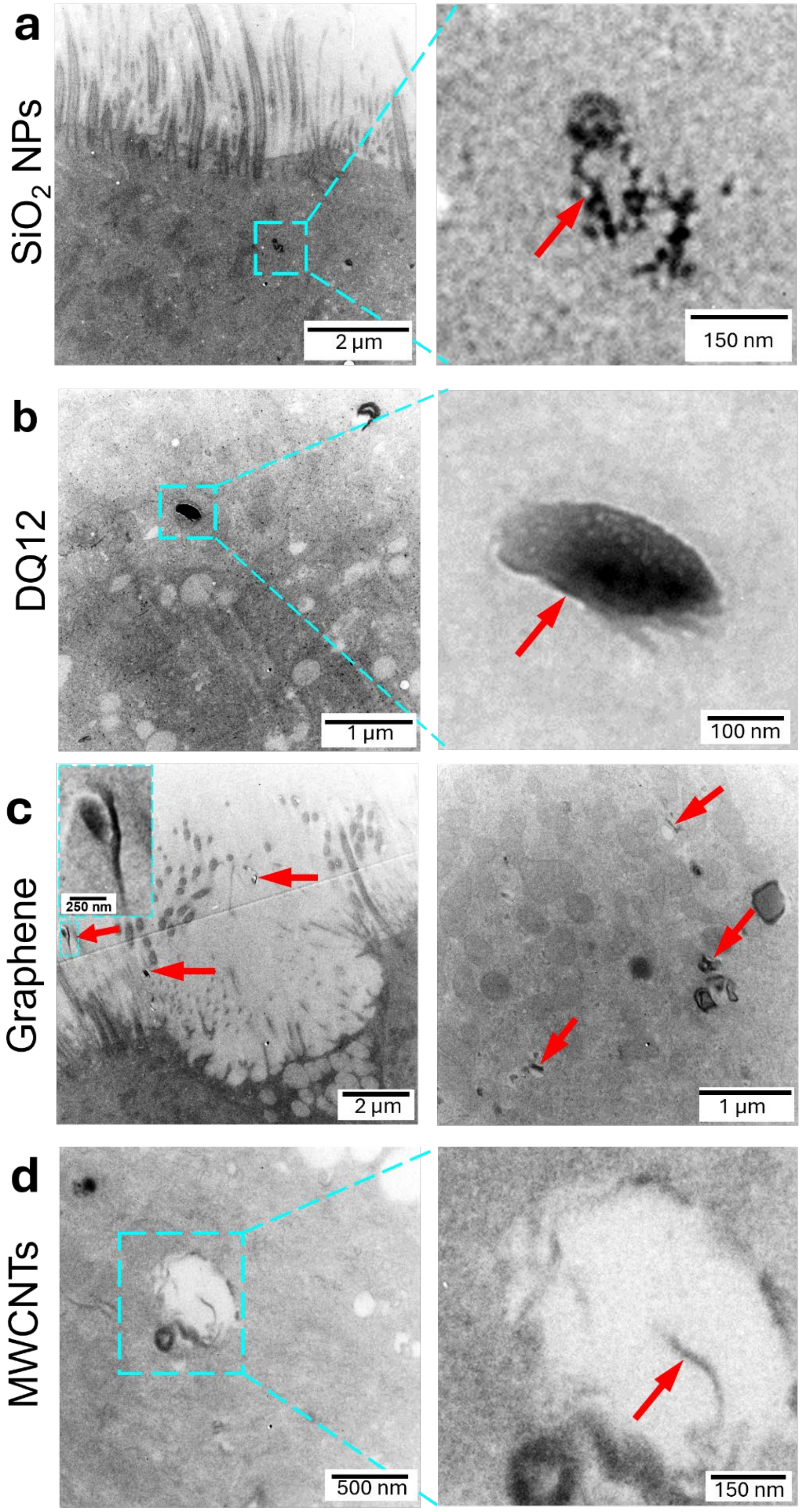

Figure S5: Cellular uptake of materials rarely found in pHBE cell cultures after 4 days of repetitive exposure (10 µg/cm²). TEM images showing (a) SiO_2 NPs, (b) DQ12, (c) graphene, and (d) MWCNTs in the cells.

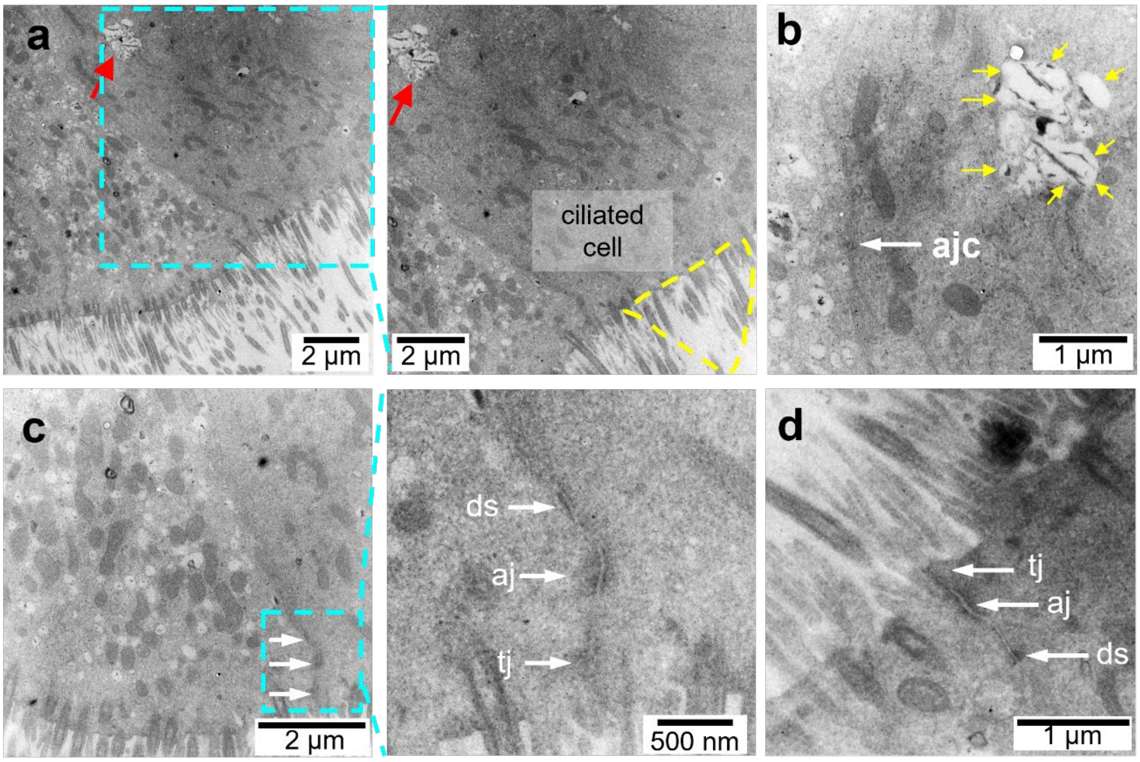

Figure S6: TEM images of pHBE cell cultures exposed to SiC nanowires (NWs) after 4 days. (a) SiC NWs accumulation (red arrows) was evident in ciliated cells as highlighted with the dashed yellow line indicating cilia on the cell membrane. (b) SiC NWs were localized in different endosomal vesicles (enclosing membranes indicated with yellow arrows), which appear to be fusing together from their spatial proximity. Apical junctional complex (ajc, white arrow) is visible on the left side. (c-d) Intact intercellular apical junctional complex, including tight junctions (tj), adherent junctions (aj), and desmosomes (ds) were visible in the epithelial layer, indicated by white arrows.

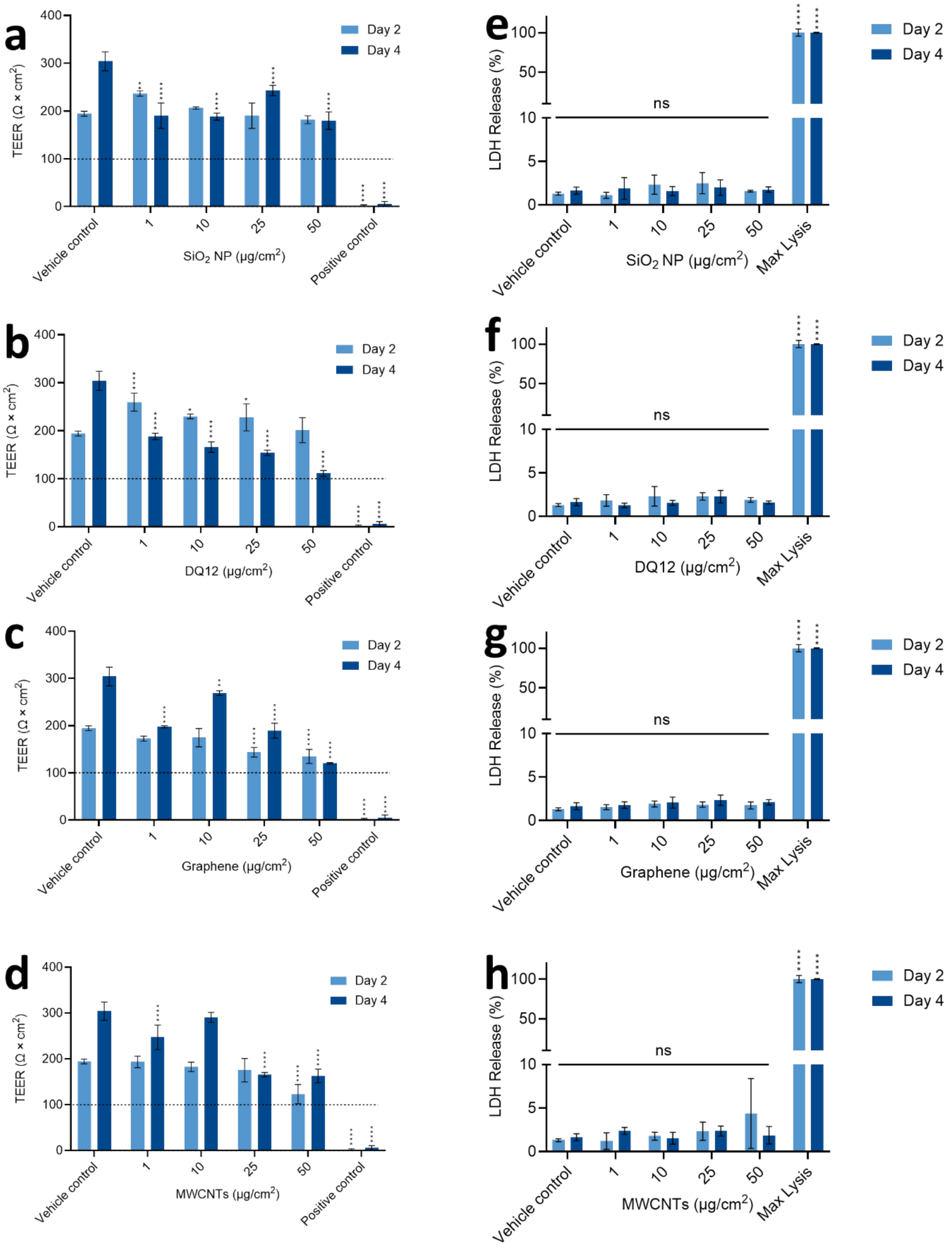

Figure S7: Cytotoxicity of materials in pHBE cell cultures after 2 and 4 days of repetitive exposure. TEER value of the cell cultures after exposure to (a) SiO_2 NPs, (b) DQ12, (c) graphene nanosheets, and (d) MWCNTs. LDH release from the cells after exposure to (e) SiO_2 NPs, (f) DQ12, (g) graphene, and (h) MWCNTs. Data presented as mean ± SD (n=3). *p*-value was calculated by applying ordinary One-way ANOVA and Dunnett's multiple comparison test for *post hoc* analysis. $*p < 0.05$ was considered statistically significant with respect to the control. $p > 0.05$ was considered as not statistically significant (ns).

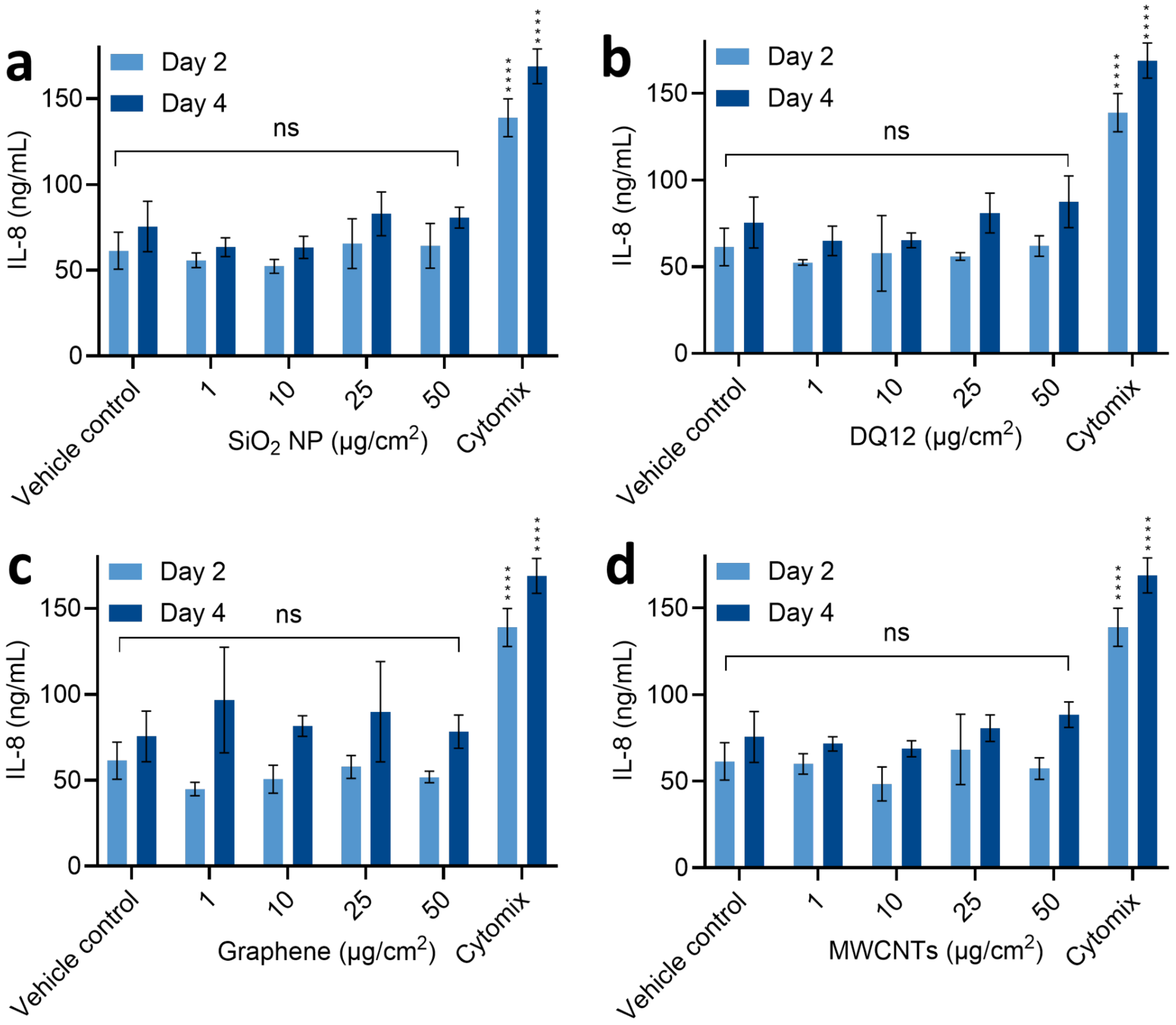

Figure S8: IL-8 release from pHBE cell cultures after 2 and 4 days of repetitive exposure. (a) SiO_2 NPs, (b) DQ12, (c) graphene, and (d) MWCNTs. Cytomix™ was used as the positive control. Data presented as mean ± SD (n=3). p-value was calculated by applying ordinary One-way ANOVA and Dunnett's multiple comparison test for *post hoc* analysis. *$p < 0.05$ was considered statistically significant with respect to the control. $p > 0.05$ was considered not statistically significant (ns).

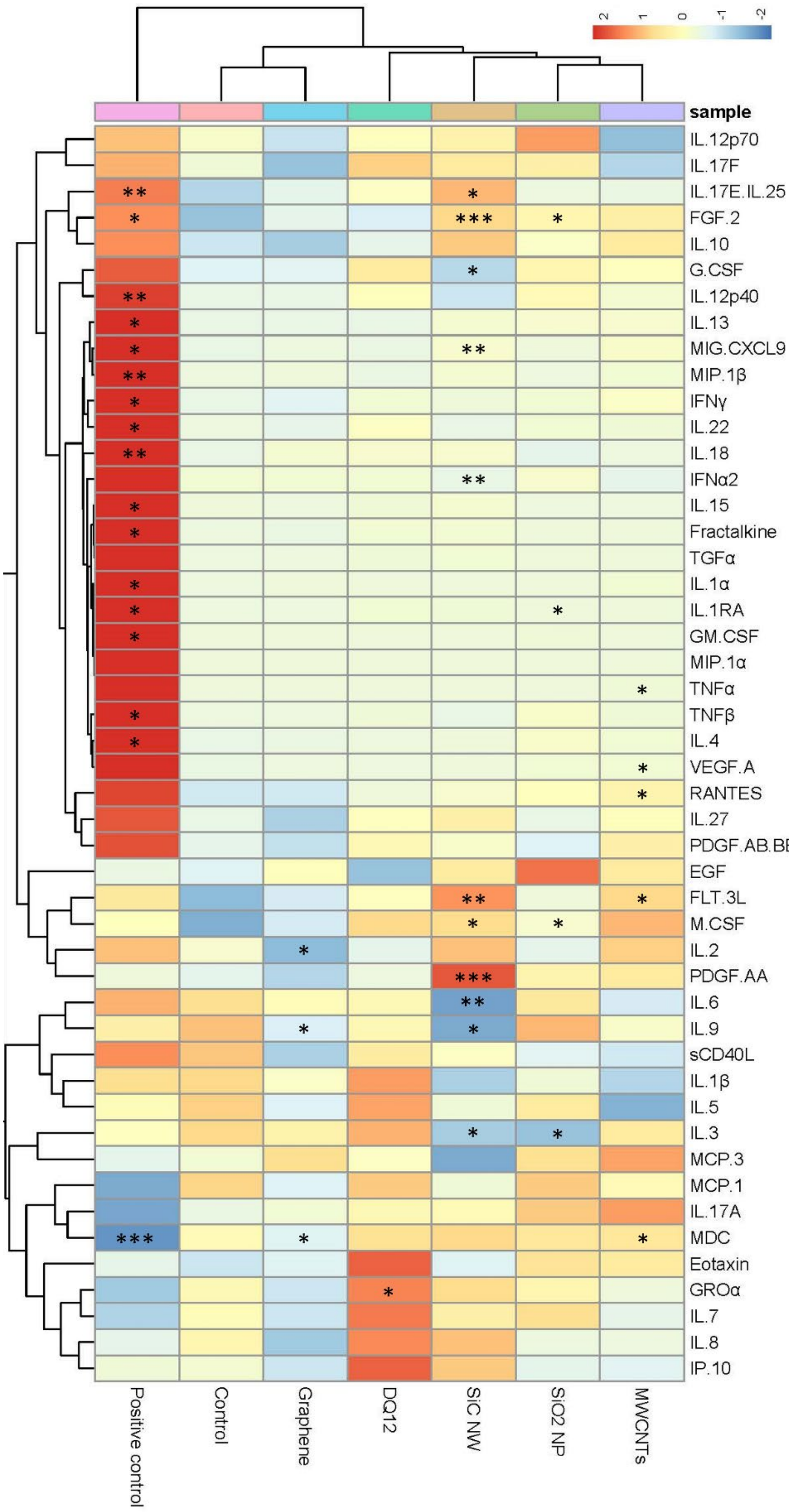

Figure S9: Heat map with hierarchical clustering shows global cytokine-chemokine response after day 2 of exposure to the indicated materials. 0.05% BSA in 0.9% NaCl solution was used as a negative control and Cytomix™ was used as a positive control. *p*-value was calculated by applying the unpaired Welch's t-test. *$p < 0.05$, **$p < 0.01$, ***$p < 0.001$ were considered statistically significant with respect to the control. $p > 0.05$ was considered as not statistically significant.

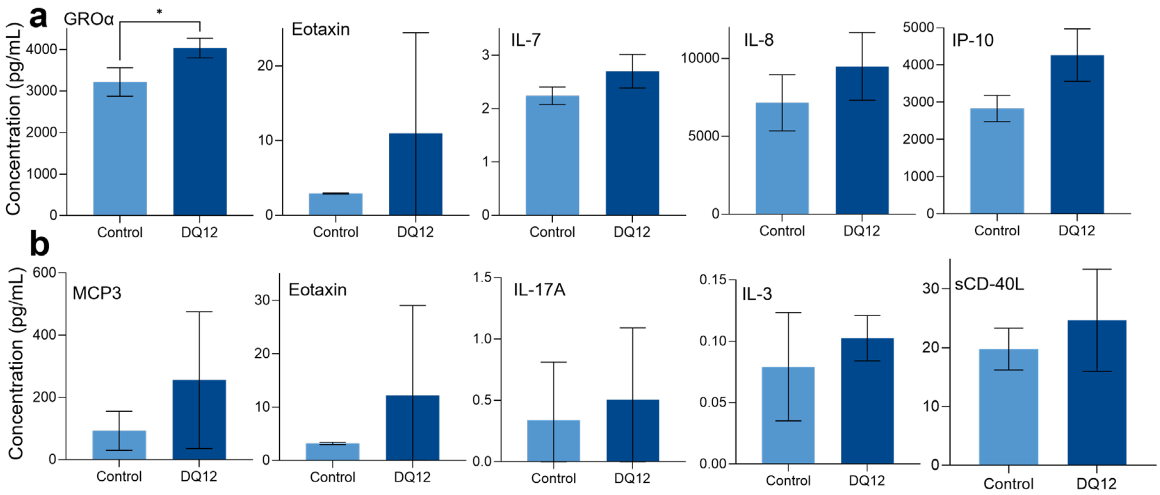

Figure S10: Cytokine-chemokine responses in pHBE cell cultures after 2 and 4 days of exposure to quartz DQ12. (a-b) The most affected cytokines after quartz DQ12 exposure on day 2 (a), and day 4 (b). Data are presented as mean ± SD (n=3). *p*-value was calculated with unpaired Welch's t-test. $*p < 0.05$ was statistically significant with respect to the vehicle control (cells exposed to 0.05% BSA in 0.9% NaCl solution).
